# Early functional changes and plasma GFAP in Swedish families with Autosomal Dominant Alzheimer’s disease mutations

**DOI:** 10.1038/s41398-026-03829-6

**Published:** 2026-01-27

**Authors:** Emma S. Luckett, Mariola Zapater-Fajari, Ove Almkvist, Charlotte Johansson, Konstantinos Chiotis, Marco Bucci, Anders Wall, Nicholas J. Ashton, Kaj Blennow, Henrik Zetterberg, Elena Rodriguez-Vieitez, Caroline Graff, Agneta Nordberg

**Affiliations:** 1 Department of Neurobiology, Care Sciences and Society, Centre for Alzheimer Research, Division of Clinical Geriatrics, Karolinska Institutet, Blickagången 16, SE-14183 Huddinge, Sweden; 2 Theme Inflammation and Aging, Karolinska University Hospital, Hälsovägen, SE-14186 Stockholm, Sweden; 3 Department of Neurobiology, Care Sciences and Society, Center for Alzheimer Research, Division of Neurogeriatrics, Karolinska Institutet, Visionsgatan 4, SE-17164 Stockholm, Sweden; 4 Turku PET Centre, University of Turku, Turku University Hospital and Åbo Akademi University, 20251 Turku, Finland; 5 Department of Surgical Sciences, Section of Molecular Imaging & Medical physics, Uppsala University, 75185 Uppsala, Sweden; 6 PET Centre, Uppsala University Hospital, 75185 Uppsala, Sweden; 7 Department of Psychiatry and Neurochemistry, Institute of Neuroscience and Physiology, Sahlgrenska Academy, University of Gothenburg, SE- Wallinsgatan 6, 43141 Mölndal, Sweden; 8 Institute of Psychiatry, Psychology & Neuroscience, King’s College London, Maurice Wohl Clinical Neuroscience Institute, 5 Cutcombe Rd, London, SE5 9RX UK; 9 NIHR Biomedical Research Centre for Mental Health & Biomedical Research Unit for Dementia at South London & Maudsley NHS Foundation, 16 De Crespigny Park, London, SE5 8AF UK; 10 Clinical Neurochemistry Laboratory, Sahlgrenska University Hospital, Klin Neurokemi Lab Hus V3, SE-43180 Mölndal, Sweden; 11 Department of Neurodegenerative Disease, UCL Institute of Neurology, Queen Square, London, WC1N 3BG UK; 12 Hong Kong Center for Neurodegenerative Diseases, Clear Water Bay, Units 1501-1502, 1512-1518, 15/F Building 17W, 17 Science Park W Ave, Science Park, Hong Kong, China; 13 Wisconsin Alzheimer’s Disease Research Center, University of Wisconsin School of Medicine and Public Health, University of Wisconsin-Madison, 600 Highland Ave J5/1 Mezzanine, Madison, WI 53792 USA; 14 Department of Pathology and Laboratory Medicine, School of Medicine and Public Health, University of Wisconsin-Madison, 53792-2420 Madison, WI USA; 15 Centre for Brain Research, Indian Institute of Science, C.V. Raman Avenue, Bangalore, 560 012 India; 16 Unit for Hereditary Dementia, Karolinska University Hospital-Solna, Karolinska Vägen 22, SE-171 64 Solna, Sweden

**Keywords:** Molecular neuroscience, Diagnostic markers, Clinical genetics

## Abstract

We aimed to understand longitudinal associations between Alzheimer’s disease (AD) biomarkers in Autosomal Dominant AD (ADAD) across estimated years to symptom onset (EYO). Forty-five individuals (19 mutation carriers [EYO = −7.9 ± 11.7 years, *APP* N = 11; *PSEN1* N = 8]) from Swedish ADAD families participated. All received baseline 18F-Flurodeoxyglucose (FDG) PET and cognitive testing, and a subset (N = 26) plasma glial fibrillary acidic protein (GFAP) measurement. Follow-up data collection (including 106 FDG scans) was performed over 7.4 ± 6.4 years (visits ranged from 1–5, EYO = −25.8 to +10.3 years in mutation carriers). Mixed effects models were applied to determine longitudinal associations. *APP* and *PSEN1* mutation carriers showed different FDG uptake profiles from EYO = −20 to −10 years, with a hypermetabolism before hypometabolism in *PSEN1* mutation carriers. Early increases in plasma GFAP were primarily related to subcortical FDG decreases and cognitive changes in *APP* mutation carriers compared to non-carriers. We provide evidence for gene-dependent biomarker trajectories in ADAD.

## Introduction

Current research studies and clinical trials are moving towards understanding AD early in its continuum prior to cognitive decline, to allow for timely therapeutic intervention to give the best prognosis. Although there are proposed time courses of AD biomarkers and pathology [1, 2], whereby pathological changes occur prior to the onset of cognitive decline, the changes in neuronal dysfunction and cognitive decline are still largely unknown in Autosomal Dominant AD (ADAD), given this has not been systematically assessed in ADAD over large time intervals. Therefore, it is important to delve into the longitudinal course of AD biomarkers and how they relate to cognitive decline. In this sense, the study of ADAD individuals provides valuable insights into biomarker changes along the disease continuum, due to the predictable age of symptom onset. Calculating the estimated years to symptom onset (EYO) allows for tracking of the disease from the earliest stages, placing biomarker changes in a temporal order. Moreover, individuals with ADAD typically develop symptoms at an earlier age compared to sporadic AD, thus eliminating certain confounding factors associated with aging. Thus, studies in ADAD cohorts allow for a clearer visualization of biomarker changes solely related to AD [3].

The investigation of ADAD individuals has evidenced that cognition starts to decline 10–15 years before expected symptom onset in specific tests, mainly in episodic memory [3–5], where cognitive phenotypes have also been shown to differ between ADAD pathogenic mutations [4]. An open question is whether pathogenic-mutation-driven cognitive differences are explained by different biomarker trajectories across the disease continuum. This decline in cognitive function in vivo can be measured through ^18^F-Flurodeoxyglucose (FDG) PET uptake as a proxy of neurodegeneration [6]. Previous evidence from ADAD individuals suggests decreased FDG uptake around 10 years before mean age of symptom onset [3, 7]. However, the time-consuming nature of cognitive tests and high cost of PET imaging have led to the emergence of new fluid biomarkers in the field.

Glial fibrillary acidic protein (GFAP) is considered a promising fluid biomarker for neurodegenerative disorders such as AD, multiple sclerosis, and frontotemporal dementia [8, 9]. Additionally, changes in GFAP may occur 10 years before diagnosis in sporadic AD [10], as well in ADAD [11–13], and can be predictive of future conversion to AD [10, 11]. Post mortem brain studies have shown GFAP is upregulated in reactive astrocytes and has been suggested to be a marker of A*β*-related reactive astrogliosis [14, 15], which subsequently drives neurodegeneration in AD observed using FDG PET. However, it should be acknowledged that although FDG PET uptake primarily reflects neurodegeneration, it can also be influenced by glial cells, providing an indirect measure of astrocyte and microglia activity [16]. This has been previously demonstrated in the literature, with studies showing that glial activation can drive changes in FDG uptake, especially in neurodegenerative disorders [17, 18]. Recent studies have elucidated the importance of the tripartite synapse, which emphasizes the important role of astrocytes in supporting synaptic activity and neuronal function [19]. Additionally, the astrocyte-neuron lactate shuttle (ANLS) is a critical mechanism in which astrocytes supply lactate to neurons as an energy source during periods of high neuronal activity [20], which is reflected in FDG PET signals. Given the fact that GFAP is considered a marker of astrocytic activation, and FDG PET reflects both neuronal and glial metabolic activity, a further understanding of the interplay between these two biomarkers is essential for assessing early functional changes in AD. Broadly, we aimed to evaluate the longitudinal trajectories of plasma GFAP, glucose metabolism measured with FDG PET, and cognition, and test their longitudinal associations. Specifically, we aimed to investigate differences between different brain regions (subcortical and cortical), multiple cognitive domains, and plasma GFAP in a gene-dependent manner. We hypothesize that a greater decrease of FDG PET uptake across EYO will be related to a steeper increase in plasma GFAP levels in mutation carriers compared to non-carriers, which will be reflected by cognitive decline. Furthermore, we hypothesize that there will be differences in the relationships between biomarkers depending on mutation carriership.

## Materials and methods

### Study participants

Individuals from Swedish families with known ADAD mutations are part of an ongoing longitudinal study at Karolinska University Hospital Huddinge that began in 1993. Forty-five of these participants were included in the present study given availability of data of interest: 19 mutation carriers (*APP Artic*_E693G_ (*AβPP*_arc_) N = 3; *APP Swedish*_KM670/671NL_ (*APP*_swe_) N = 8; *PSEN1*_H163Y_ N = 6; *PSEN1*_M146V_ N = 2) and 26 non-carriers. All individuals underwent baseline FDG PET and cognitive testing, and a subset (N = 26) received plasma GFAP concentration measurement (10 mutation carriers: 5 *PSEN1*_H163Y_, 2 *APP*_swe_, 3 *AβPP*_arc_ and 16 non-carriers). Furthermore, individuals received follow-up data collection of all variables over an interval of 7.4 ± 6.4 years, with the number of total visits ranging from one to five. Individuals with more than one visit were 14 mutation carriers (6 *PSEN1*_H163Y_, 2 *PSEN1*_*M146V*_, 5 *APP*_swe_, 1 *AβPP*_arc_) and 18 non-carriers. For plasma GFAP, 8 mutation-carriers had longitudinal data available (5 *PSEN1*_H163Y_, 2 *APP*_swe_, 1 *AβPP*_arc_) and 12 non-carriers. Participants in this subgroup did not significantly differ from the full cohort, except for follow-up time interval, which was due to the more limited number of collected samples (data not shown).

For each specific mutation, the average age of symptom onset was defined as the average age at which clinical symptoms first appeared. These correspond to: *AβPP*_arc_ = 56 ± 3 years; *APP*_swe_ = 54 ± 5 years; *PSEN1*_H163Y_ = 52 ± 7; *PSEN1*_M146V_ = 36 ± 3. From this, estimated years from symptom onset (EYO) was calculated for each individual as the relative difference from this age. This was calculated for both mutation carriers and non-carriers identically.

To maintain confidentiality, limited information about these participants is provided. Furthermore, individuals present in this study were recruited based on available data from rare mutations, and thus the case-control design is not matched due to limited families with the associated mutations.

### Neuropsychological evaluation

A similar approach used in Almkvist et al. was used for the assessment of cognitive function [4]. Briefly, seven cognitive tests were used to address five different cognitive domains. The Rey Auditory Verbal Learning (RAVL learning) was used to assess verbal episodic memory; retention was measured using the delayed recall of the RAVL (RAVL retention); and the delayed recall of the Rey-Osterrieth Complex Figure (RO retention) was used to assess visuospatial episodic memory [21]. Executive function was assessed with both the Digit Symbol test of the Wechsler Adult Intelligence Scale Revised [22, 23], and Trail Making Test B (TMT-B) [21]. Finally, short term memory/attention was measured using Digit Span Forward and Trail Making Test A (TMT- A) [21]. All raw scores were converted to *Z*-scores using non-carriers (N = 26) as the reference group.

### ^18^F-Fluorodeoxyglucose PET acquisition and processing

Participants underwent dynamic FDG PET acquisition at the Uppsala PET Centre, Uppsala University, between 1993–2013, which were processed as previously described [7, 12]. Briefly, images were processed using Statistical Parametric Mapping Version 12 (Wellcome Trust Centre for Neuroimaging, London, UK, http://www.fil.ion.ucl.ac.uk/spm) running on MATLAB R2023a (Mathworks, Natick, MA, USA), using the acquisition window 40–60 min post injection. Normalization to Montreal Neurological Institute space was achieved using an FDG template [24]. The pons was used as a reference region to generate standardized uptake value ratios (SUVRs) from the summed PET images in several regions of interest defined using composite ROIs based on a grey matter version of the Hammers Atlas: frontal, temporal, parietal, occipital, and posterior cingulate cortices, a temporoparietal meta-region of interest, thalamus, hippocampus, amygdala, caudate, pallidum, and putamen subcortical areas [7, 12, 25, 26]. Given the small sample size and no a priori laterality predictions, SUVRs were calculated using data combined from both hemispheres prior to analyses.

### Plasma sampling and quantification

Whole-blood samples were collected for a subset of individuals (N = 26). Prior to 2015, samples were obtained in sodium heparin tubes; following a protocol change, samples were collected in EDTA tubes. Based on this timeline, only two of the 26 individuals had plasma collected in EDTA tubes at a single time point, and all samples were processed as previously described [12, 13]. Plasma GFAP concentrations were measured in one batch at the Clinical Neurochemistry Laboratory, Sahlgrenska University Hospital, Gothenburg, using the Quanterix Simoa^TM^ Human Neurology 4-plex A Assay (Quanterix Corporation, Billerica, MA). The lower limit of quantification and pooled coefficient of variation were 0.467 pg/ml and 12.9%, respectively. Raw GFAP concentration values were log-transformed prior to analyses to assume a normal distribution. No other plasma markers were available for analyses.

### Statistical analysis

Statistical analyses were performed in R version 4.3.1 (2023-06-16; The R Foundation for Statistical Computing; https://www.cran.r-project.org/). Scripts used to generate the results reported in this manuscript are available from the corresponding author upon reasonable request. Normality of all data was checked using Shapiro-Wilk tests, as well as normality of residuals using the *check_model* function, prior to analyses to ensure model assumptions were met. No formal a priori power calculation was performed, the sample size was determined by the availability of participants. Outliers were determined using *Grubb’s Test*. *P*-values were considered significant when < 0.05. All linear mixed effects s (LMEs) described below were performed using the R package *lme4*. Estimates and confidence intervals from statistical models are not presented in the text due to the number of models performed but can be found in the figures and/or supplementary tables. For each of the models described, all the biomarker variables available were analysed as predictor or outcome. Accordingly, this was each of the 12 FDG regions quantified (SUVRs); each of the seven individual cognitive test *Z*-scores; and log-transformed plasma GFAP concentration. For some analyses, there were missing cognitive scores due to lack of clinical availability. Due to the small sample size and this missing data, data points were excluded as necessary from the analyses rather than imputed. This corresponded to analyses involving RAVL Retention and Digit Span Forward scores.

First, LMEs were used to determine the longitudinal evolution of each biomarker separately across EYO, with either FDG, cognition, or plasma GFAP concentration as outcome. Second, LMEs were used to determine longitudinal relationships between different biomarkers, where three relationships were implemented: (i) cognitive scores with FDG SUVRs as predictor; (ii) FDG SUVRs with plasma GFAP concentration as predictor; (iii) predicted cognitive scores with plasma GFAP concentration as predictor. For each relationship, two LMEs were run: Model 1 included the interaction term variable*carrier/non-carrier, and Model 2 included the interaction term variable*APP/*PSEN1*/non-carrier. For all analyses, subject ID was included as a random effect to account for repeated measures, with baseline age, education, EYO and EYO^2^ as covariates, with EYO included as the interaction term in the analyses investigating the longitudinal evolution of individual biomarkers.

As an exploratory analysis, due to the limited sample size, we performed the same LMEs as described above in a subgroup of mutation carriers only after the removal of all non-carriers, to determine whether the trajectories of *APP* and *PSEN1* carriers significantly deviated from each other (Model 3). Due to the small number of individuals carrying specific mutations within the two mutation groups, no further stratified analyses were performed.

All the statistics described used the log-transformed plasma GFAP values, but raw GFAP values are displayed in all the figures for clarity. Note that one data point appears to be an outlier prior to log transformation, however this is not the case after, thus it is not removed from the analyses.

As an alternative to the region of interest-based analyses, we also performed voxelwise analyses using VoxelStats [27] employing the same LMEs as above with FDG as outcome and baseline age, education, EYO and EYO² as covariates, with EYO included as the interaction term (variable*carrier/non-carrier, and another with interaction term variable*APP/*PSEN1*/non-carrier).

Representative plots depict the temporoparietal and caudate ROI SUVRs or verbal episodic memory scores for consistency across analyses, however, all results are presented in the Supplementary Information.

## Results

For cohort characteristics see Table 1. Overall, individuals had a baseline age of 43.8 ± 11.8 years and 42.7 ± 12.8 years in the mutation carrier and non-carrier groups, respectively. Furthermore, the number of visits for all individuals ranged from one to five, with EYO ranging from −25.8 to +10.3 years in mutation carriers over the total time investigated. There was a variation in the number of follow-up visits due to motivation of the participants and, to some extent, discontinuation due to progression of the disease or that they passed away.

**Table 1:** Cohort characteristics.

					Overall	
	*AβPParc* (N = 3)	*APPswe* (N = 8)	*PSEN1*_*H163Y*_ (N = 6)	*PSEN1*_*M146V*_ (N = 2)	Non-carrier (N = 26)	Carrier (N = 19)
**Baseline age (years)**	59.7 (3.79)	45.5 (11.4)	34.9 (7.68)	40.8(0.70)	42.7 (12.8)	43.8 (11.8)
**Sex (male, %)**	1 (33.3%)	4 (50.0%)	6 (100%)	0(0%)	17 (65.4%)	11 (57.9%)
***APOE4*** **status (carrier, %)**	0 (0%)	4 (50.0%)	3(50%)	2(100%)	7 (26.9%)	9 (47.4%)
**Education (years)**	12.3 (3.21)	10.6 (1.85)	11.2(1.33)	9(0)	11.0 (2.39)	10.9 (1.94)
**MMSE**	30.0 (NA)	24.8 (7.26)	29.4 (1.34)	19(7.07)	28.9 (1.64)	26.1 (6.10)
**Mutation age of symptom onset (years)**	56 ± 3	54 ± 5	52 ± 7	36 ± 3	51.3 (6.79)	51.8 (5.73)
**Estimated years to symptom onset**	3.63 (3.62)	−8.48 (11.4)	−17.1(7.68)	4.83(0.70)	−8.69 (10.8)	−7.89 (11.7)
**Time interval (for participants with follow-up, years)**	2.40 (NA)	5.91 (7.24)	1.4 (7.06)	2.93 (1.74)	7.06 (5.99)	7.57 (6.97)

For all continuous measures the mean and standard deviations are shown, for categorical measures the number of individuals and the associated percentage is shown. Participants with FDG PET and cognitive data at follow-up: 6 *PSEN1*_H163Y_, 2 *PSEN1*_*M146V*_, 5 *APP*_swe_, 1 *AβPP*_arc_).

### Longitudinal evolution of individual biomarkers across EYO

**Fig. 1:**
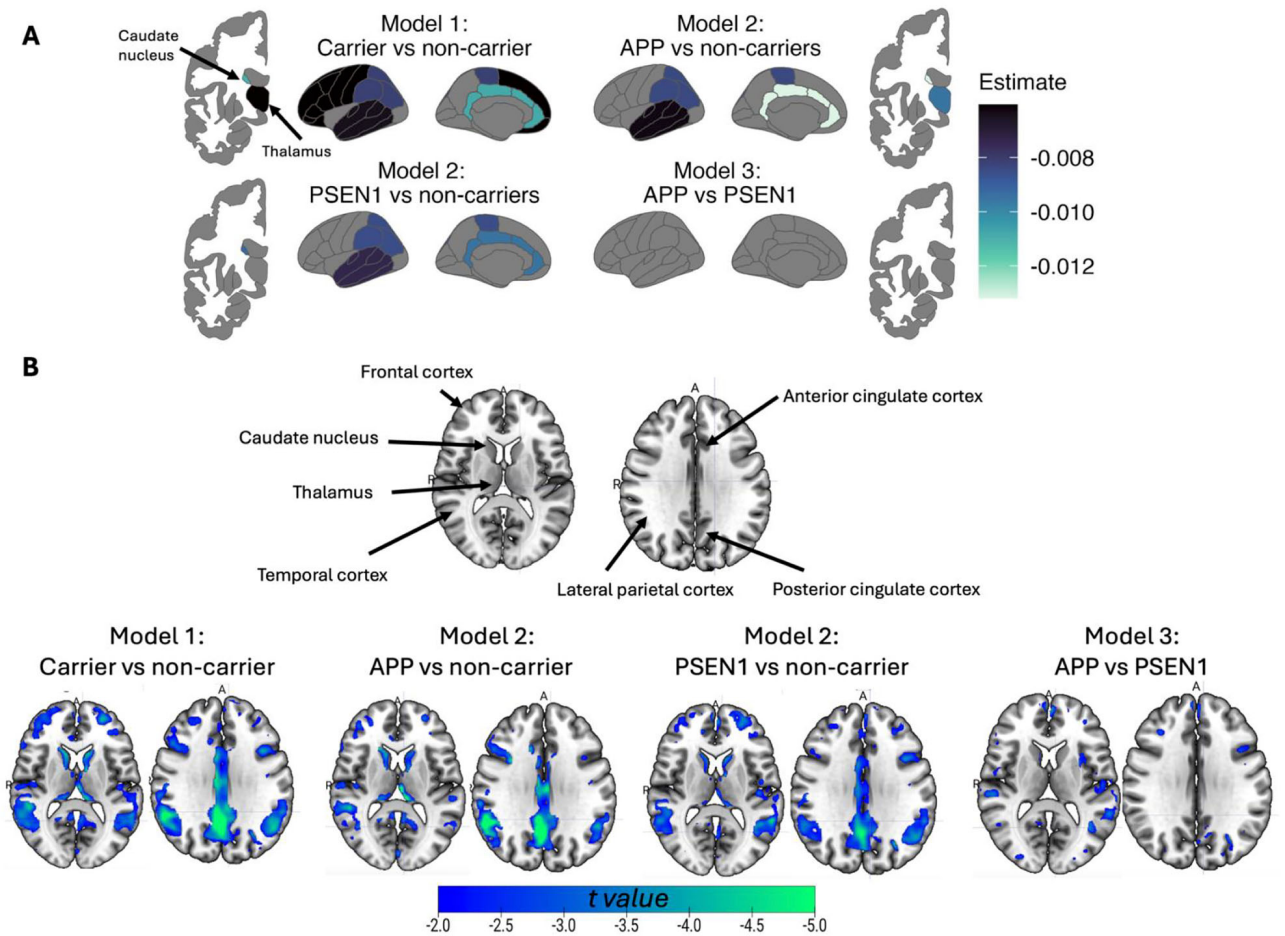
Regional longitudinal trajectories of glucose metabolism (FDG PET) across estimated years to symptom onset. Region of interest surface maps (**A**) and voxelwise surface projections (**B**) illustrate the brain regions that were significantly decreasing in glucose metabolism over the time interval analysed. For (**A**) the estimates, representing the effect, from the linear mixed effects models are depicted, where a lighter colour indicates a larger decrease in glucose metabolism over time. For (**B**) the t values, representing the significance from the voxelwise linear mixed effects models are depicted, where a lighter colour indicates a larger decrease in glucose metabolism over time.

**Fig. 2:**
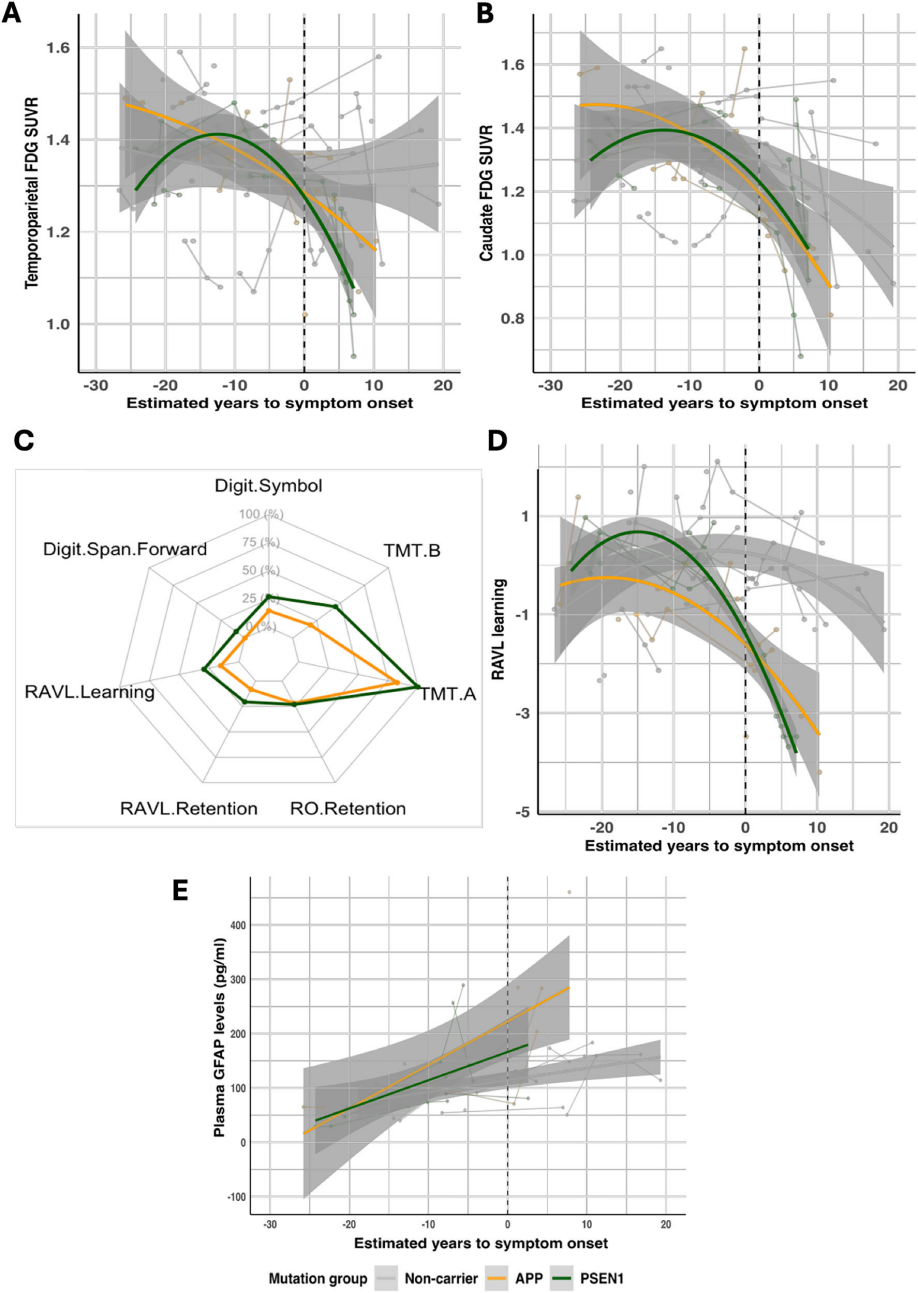
Representative longitudinal trajectories of glucose metabolism (FDG PET), cognitive outcomes, and plasma GFAP concentration. (**A**) Temporoparietal FDG SUVRs and (**B**) Caudate FDG SUVRs across estimated years to symptom onset in *APP* and *PSEN1* mutation carriers, and non-carriers. (**C**) Radar plot depicting the estimates, representing the effect, from the linear mixed effects models. The percentage represents the difference from the minimum (0%, −0.04) and maximum (100%, −0.43) estimate values, representing the smallest and largest change in a particular cognitive test. (**D**) Representative RAVL learning trajectories and (**E**) Raw plasma GFAP concentration values across estimated years to symptom onset in *APP* and *PSEN1* mutation carriers, and non-carriers. Abbreviations: SUVR = standardised uptake value ratio.

Compared to non-carriers, mutation carriers showed a significant decrease in FDG uptake across EYO for all cortical regions analysed (all *p* ≤ 0.041) except for occipital (*p* = 0.297, Fig. 1A and B, representative Fig. 2A, Supplementary Fig. 1, Supplementary Table 1). Only the thalamus (*p* = 0.045) and caudate (*p* = 0.001) for the subcortical regions showed a significant decline in FDG uptake across EYO in mutation carriers compared to non-carriers (Fig. 1A and B, representative Fig. 2B, Supplementary Table 1). Furthermore, when assessing the specific mutations, significance was observed for all brain regions except frontal and occipital for both *PSEN1* carriers compared to non-carriers (*p* = 0.065, *p* = 0.363, respectively) and *APP* carriers compared to non-carriers (*p* = 0.103 and *p* = 0.273, respectively). The caudate had a significant decline in FDG PET uptake in both mutation groups respective to non-carriers (*PSEN1* mutation carriers compared to non-carriers *p* = 0.023, *APP* mutation carriers compared to non-carriers *p* = 0.013), but only the thalamus significantly declined in FDG PET uptake in *APP* mutation carriers compared to non-carriers (*p* = 0.013, Fig. 1A and B). It can be observed from the estimates and t values in Fig. 1A and B that individuals have a decrease in FDG uptake over time, where *APP* and *PSEN1* mutation carriers become hypometabolic in similar areas but at different rates. Lastly, FDG trajectories did not significantly differ across EYO in *APP* carriers compared to *PSEN1* carriers for any of the regions analysed (all *p* ≥ 0.135). However, we observe a hypermetabolism in FDG uptake prior to hypometabolism in *PSEN1* mutation carriers at approximately EYO = −20 to −10 years, but a gradual decline in FDG uptake in *APP* mutation carriers (Representative Fig. 2A and B). When performing voxelwise analyses, results from these linear mixed effects corroborated the region of interest-based analyses in all models analysed (Fig. 1A and B).

All cognitive tests significantly declined across EYO in mutation carriers compared to non-carriers (all *p* ≤ 0.011, Supplementary Fig. 2, Supplementary Table 2). All tests significantly declined in *PSEN1* carriers compared to non-carriers (all *p* ≤ 0.006), whereas all tests except RAVL retention (trend *p* = 0.052) and Digit Span Forward (*p* = 0.151) significantly declined in *APP* carriers compared to non-carriers (remaining *p* ≤ 0.005, Fig. 2C, representative Fig. 2D). Additionally, results showed that only the trajectories across EYO of Digit Symbol (*p* = 4.31e-04) and TMT-B (*p* = 0.032) were significantly different between *PSEN1* and *APP* mutation carriers.

There was a steady increase in plasma GFAP levels in all individuals across EYO, however, longitudinal changes in plasma GFAP levels were not significantly different between mutation carriers and non-carriers (*p* = 0.126, *t* = 1.56, estimate and 95% confidence interval (CI) = 0.023 (−0.01, 0.05), Fig. 2E). This was gene dependent as *PSEN1* carriers had a significant increase in plasma GFAP across EYO compared to non-carriers (*p* = 0.026, *t* = 2.30, estimate (CI) = 0.033 (0.005, 0.06), but *APP* carriers compared to non-carriers did not (*p* = 0.111, *t* = 1.63, estimate (CI) = 0.021 (−0.004, 0.05)). There was no difference between the GFAP trajectories of *APP* and *PSEN1* carriers (*p* = 0.567, *t* = 0.582, estimate (CI) = 0.011 (−0.03, 0.05).

### Longitudinal relationships between FDG and cognition

**Fig. 3:**
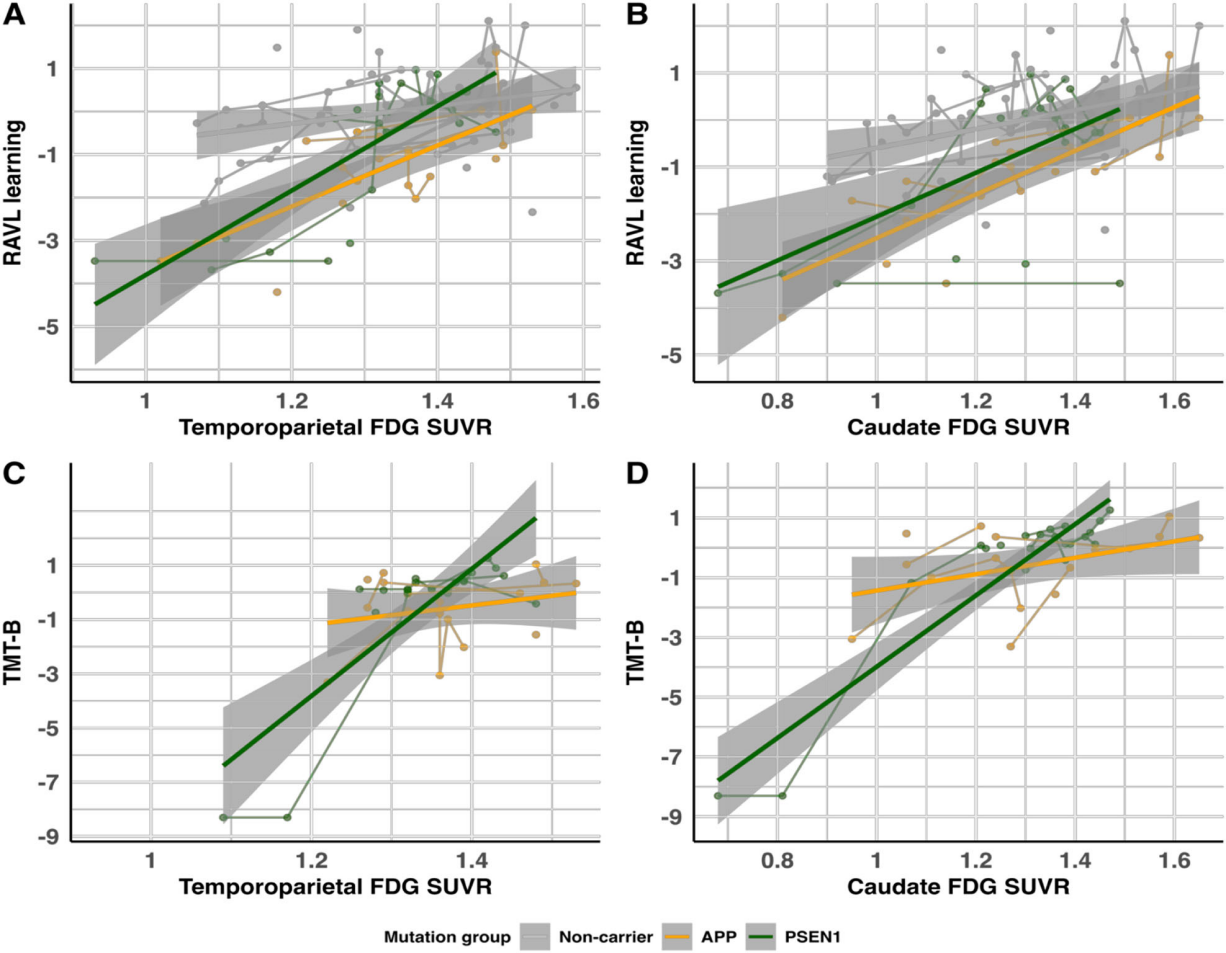
Longitudinal relationships between FDG PET and cognition. As a representation of all the data, depicted are the relationships from the significant LMEs for RAVL learning scores (episodic memory) with (**A**) temporoparietal FDG SUVRs and (**B**) caudate FDG SUVRs for *APP* and *PSEN1* mutation carriers and non-carriers, and TMT-B scores with (**C**) temporoparietal FDG SUVRs and (**D**) caudate FDG SUVRs for *APP* and *PSEN1* mutation carriers. Abbreviations: SUVR = standardised uptake value ratio.

Supplementary Table 3 depicts the relationships between cortical regional FDG uptake and the different cognitive tests in mutation carriers compared to non-carriers. In mutation carriers, steeper FDG temporoparietal decline was found to be associated with steeper decline in all cognitive tests (*p* ≤ 0.005) except a trend in RAVL retention (*p* = 0.050) and a lack of association with Digit Span Forward (*p* = 0.676) compared to non-carriers (representative plot in Fig. 3A). Furthermore, frontal, temporal and parietal FDG SUVRs were significantly associated with all tests (*p* ≤ 0.042) except Digit Span Forward (*p* = 0.499, *p* = 0.518, *p* = 0.789, respectively) in mutation carriers compared to non-carriers. The parietal region was not significantly associated with RAVL retention (*p* = 0.063) in mutation carriers compared to non-carriers. The decline in FDG uptake in the occipital region was only related to a decline in Digit Symbol (*p* = 0.007) and TMT-A (*p* ≤ 3.63e-05) scores in mutation carriers compared to non-carriers (remaining *p* ≥ 0.299). Lastly, decline in FDG uptake in the posterior cingulate was only significantly associated with a decline in RO retention (*p* = 2.67e-04), Digit Symbol (*p* = 0.002), TMT-B scores (*p* = 0.001), and TMT-A (*p* = 4.54e-05) in mutation carriers compared to non-carriers (remaining *p* ≥ 0.065).

When analysing longitudinal associations between subcortical FDG uptake and cognitive outcomes (Supplementary Table 4), the decline in FDG uptake in the thalamus and caudate was related to a decline in all cognitive outcomes (*p* ≤ 0.024, *p* ≤ 0.006, respectively), except RAVL retention (*p* = 0.179, *p* = 0.253, respectively) and Digit Span Forward (*p* = 0.485, *p* = 0.375, respectively) in mutation carriers compared to non-carriers. The decline in FDG uptake in the hippocampus and putamen was only related to a decline in TMT-A in mutation carriers compared to non-carriers (*p* = 1.39e-03, *p* = 9.85e-04, remaining *p* ≥ 0.204). FDG decline in the amygdala was significantly associated with a decline in Digit Symbol and TMT-A (*p* = 0.014, *p* = 1.38e-05, remaining *p* ≥ 0.098) in mutation carriers compared to non-carriers. There were no significant associations between declining glucose metabolism in the pallidum and cognitive outcomes.

The significant associations that were described above were largely driven by *PSEN1* mutation carriers compared to non-carriers, rather than *APP* mutation carriers compared to non-carriers, as can be observed in Supplementary Tables 5, 6, 7, and 8. Supplementary Tables 5 and 6 (representative Fig. 3) show that *APP* mutation carriers tended to have decreases in FDG uptake over time that were not significantly associated with a decline in cognitive outcomes. Instead, *PSEN1* mutation carriers compared to non-carriers had a significant association in their decline in FDG uptake and cognition for almost all brain regions and cognitive tests assessed (Supplementary Tables 7 and 8, representative Fig. 3). Nonetheless, when comparing *PSEN1* with *APP* mutation carriers, significant differences were found for TMT-B decline associated with a decline in FDG uptake for all cortical brain regions assessed (Fig. 3C, Supplementary Table 9), as well as for Digit Symbol with the temporal FDG uptake. On the other hand, there were more significant differences in *PSEN1* compared to *APP* mutation carriers when analysing the relationship between subcortical FDG PET uptake and cognitive outcomes (Supplementary Table 10). The thalamus and caudate FDG SUVRs were significantly associated with a decline in RO Retention, Digit Symbol, and TMT-B scores in *PSEN1* compared to *APP* mutation carriers (Representative Fig. 3D). TMT-B scores were also significantly associated with FDG uptake decline in the amygdala, whereas TMT-A scores were significantly associated with FDG uptake decline in the hippocampus, in *PSEN1* compared to *APP* mutation carriers.

### Longitudinal relationships between FDG and plasma GFAP

**Fig. 4:**
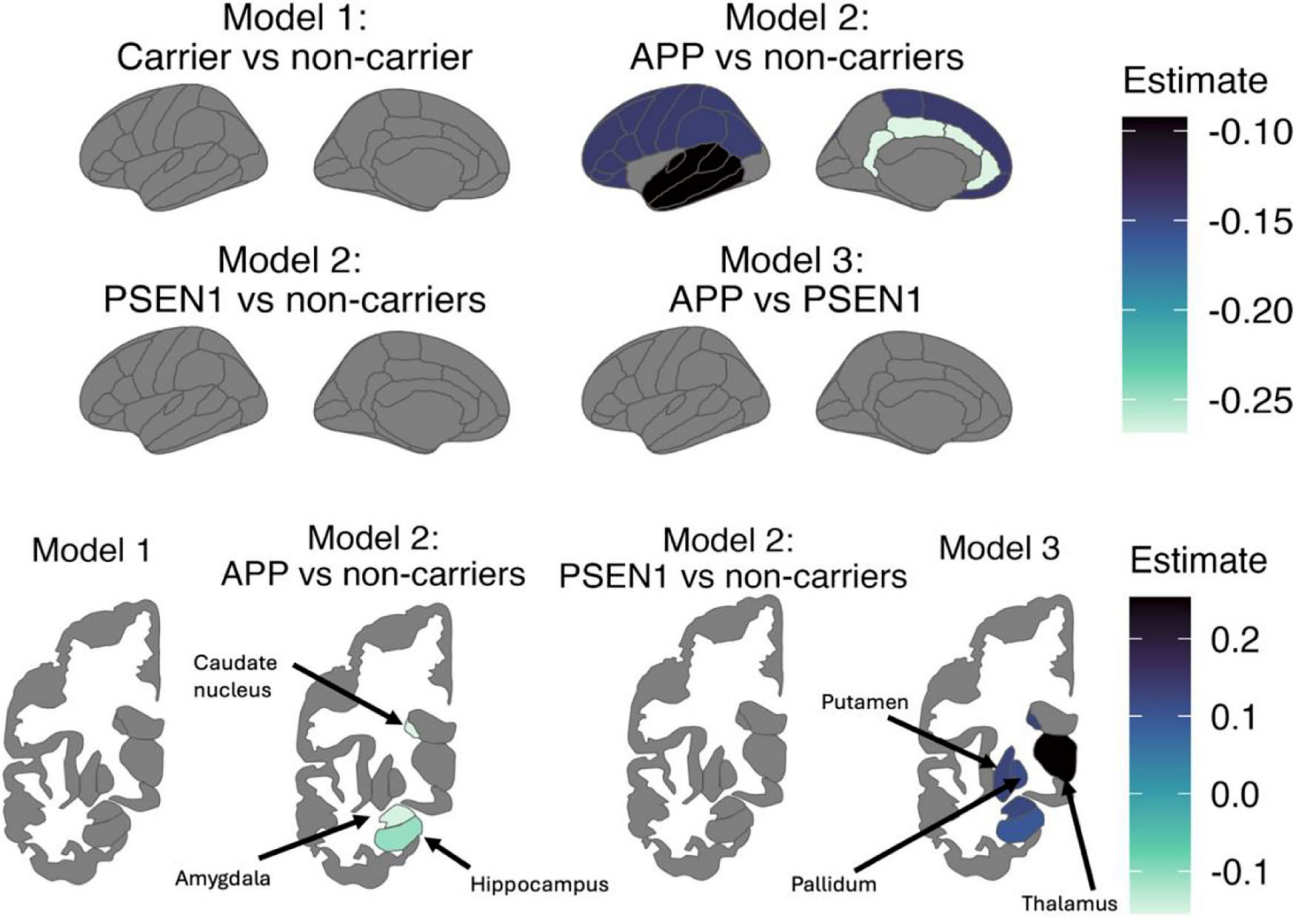
Regional longitudinal associations between glucose metabolism (FDG PET) and plasma GFAP across estimated years to symptom onset. The surface maps illustrate the brain regions that had a significant association between glucose metabolism and plasma GFAP over the time interval analysed. The estimates, representing the effect, from the linear mixed effects models are depicted, where a lighter colour indicates a larger decrease in glucose metabolism with increasing plasma GFAP concentration over time.

**Fig. 5:**
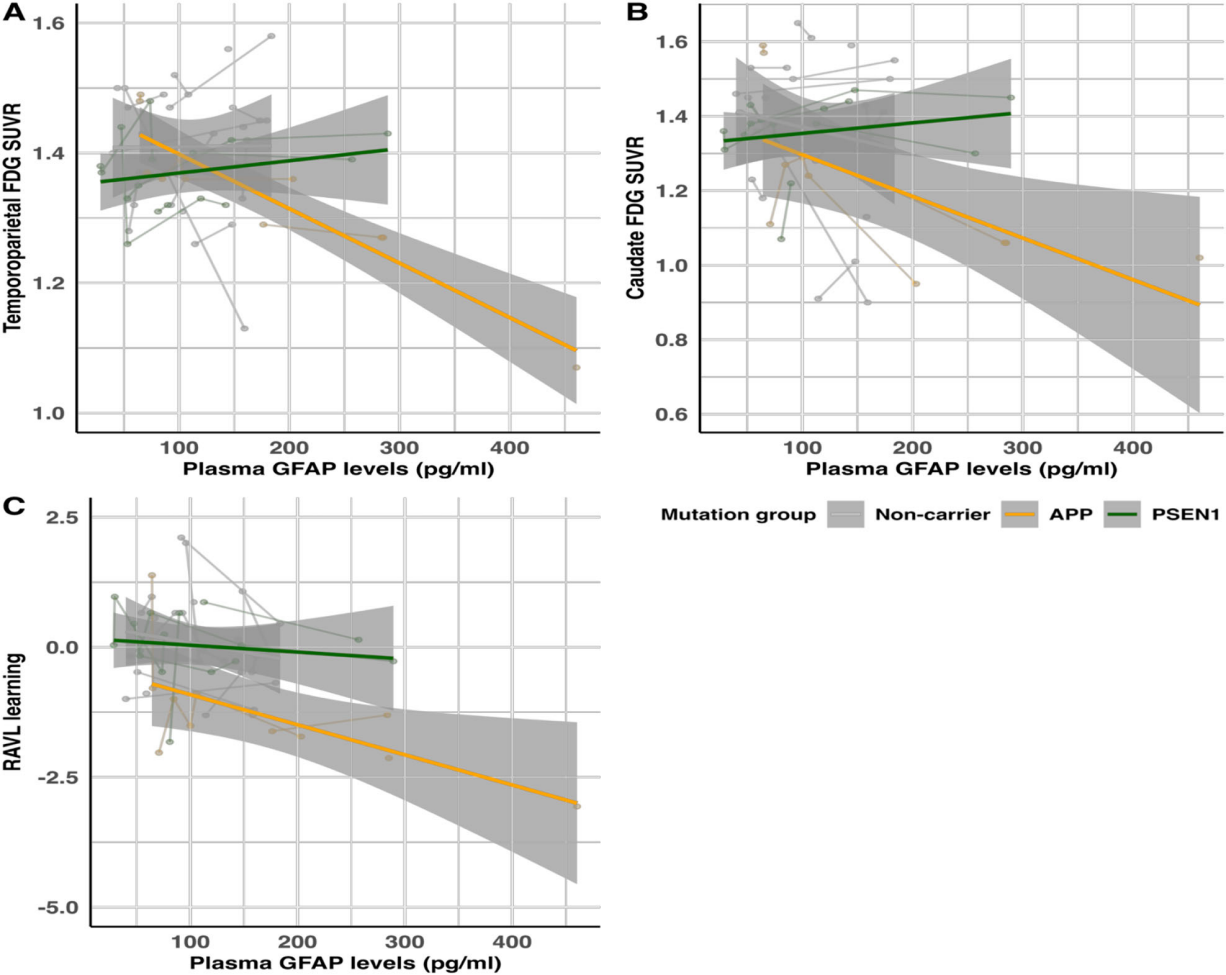
Representative longitudinal associations between plasma GFAP concentration and glucose metabolism (FDG PET) or cognitive test scores. Representative associations of plasma GFAP concentration with (**A**) temporoparietal FDG SUVRs, (**B**) caudate FDG SUVRs, and (**C**) RAVL learning scores for *APP* and *PSEN1* mutation carriers, and non-carriers. Abbreviations: SUVR = standardised uptake value ratio.

The association between plasma GFAP levels and FDG PET uptake was not moderated by mutation carriership for any of the cortical brain regions analysed (all *p* ≥ 0.076, Supplementary Table 11, Supplementary Fig. 3). However, this was gene-dependent, as *APP* mutation carriers did show an increase in plasma GFAP with a decrease in cortical FDG uptake compared to non-carriers for all brain regions (all *p* ≤ 0.025) except for the occipital region (*p* = 0.123), as observed in Fig. 4 and representative Fig. 5A. Furthermore, *APP* mutation carriers showed an increase in plasma GFAP with decreasing subcortical FDG uptake compared to non-carriers for the hippocampus, amygdala, and caudate (*p* = 0.004, *p* = 0.0002, *p* = 0.027, respectively, rest *p* ≥ 0.067, Fig. 4, representative Fig. 5B). *PSEN1* mutation carriers did not significantly differ from non-carriers (all *p* ≥ 0.181, Fig. 4). However, *APP* mutation carriers did significantly differ from *PSEN1* mutation carriers for all subcortical regions analysed (subcortical regions *p* ≤ 0.048, Fig. 4).

### Longitudinal relationships between plasma GFAP and cognition

A longitudinal increase in plasma GFAP was related to a significant decline only in RAVL learning scores in mutation carriers compared to non-carriers (*p* = 0.035, Supplementary Table 12, representative Fig. 5C, Supplementary Fig. 4). There was a gene-dependent relationship, where an increase in plasma GFAP levels was related to a decline in most cognitive tests in *APP* mutation carriers compared to non-carriers (all *p* ≤ 0.020), except for RAVL retention (*p* = 0.156) and Digit Span Forward (*p* = 0.933), which was not observed in *PSEN1* mutation carriers compared to non-carriers (all *p* ≥ 0.267). Only TMT-A was significantly associated with increasing plasma GFAP in *APP* compared to *PSEN1* mutation carriers (*p* = 0.036, rest *p* ≥ 0.084, Supplementary Table 12).

## Discussion

The main findings of our study are gene-dependent longitudinal differences in the trajectories of AD biomarkers and their associations. First, we showed that cerebral glucose hypermetabolism in *PSEN1* mutation carriers, reflected by an increase in cognitive outcomes prior to hypometabolism, was occurring around −20 to −10 years before expected symptom onset, which was not observed in *APP* mutation carriers. Only subcortical regions of caudate and thalamus showed a significant decrease in FDG uptake in mutation carriers compared to non-carriers, which was mainly driven by *APP* carriers. Secondly, there was a steady increase across EYO of plasma GFAP, where *APP* carriers showed an early increase in plasma GFAP in parallel to the declining FDG uptake in subcortical regions, which was not observed in *PSEN1* carriers. Thirdly, we showed that an increase in plasma GFAP levels was related to a decline in cognition in *APP* carriers, not observed in *PSEN1* carriers. These results provide further evidence for gene-dependent ADAD heterogeneity, suggesting different biomarker trajectories for *PSEN1* carriers.

Changes in glucose metabolism as assessed by FDG PET were similar for all brain regions assessed for both *APP* and *PSEN1* mutation carriers. Notable is that we observed a hypermetabolism in *PSEN1* mutation carriers approximately −20 to −10 years before expected symptom onset, which has previously been reported in ADAD [28] and sporadic AD [29, 30]. This hypermetabolism coincides with an increase in cognitive scores in these individuals. This may be a neuronal compensatory mechanism resulting in increased metabolism [31, 32], with a potential learning effect for cognition [33], prior to a threshold being reached, after which glucose hypometabolism and cognitive decline begin. The integrated ANLS hypothesis may play an important role where, in high-energy-demanding situations, astrocytes supply neurons with lactate as an alternative energy source [34]. This suggests that the FDG PET signal might largely reflect glucose consumption in astrocytes rather than neurons as suggested by Magistretti and Pellerin [35], and Zimmer et al. [18]. A “first wave” increase of astrogliosis measured as increased MAO-B activity by 11[C]deprenyl (DED) PET has been observed in sporadic MCI patients as well as presymptomatic ADAD mutations carriers [7, 36–38]. A longitudinal decline in DED binding in ADAD mutation carriers, which parallelled a progressive hypometabolism decrease in FDG uptake [39], supports the view of the central role of astrocytes in AD. This assumption challenges the use of FDG PET as a sole neurodegenerative marker in the ATN framework given it is influenced by astrocytic activity.

Some brain regions and cognitive outcomes appear to decline earlier than others, for example temporoparietal FDG uptake and episodic memory appear to decline approximately −15 years before expected symptom onset. This is in line with recent publications that suggest episodic memory is one of the first cognitive domains that starts to decline in (AD)AD [4, 40–43]. Lastly, these increases were not observed in *APP* mutation carriers, since these individuals appear to have a gradual decline in glucose metabolism that is reflected by a gradual decline in cognition. Similarly, plasma GFAP levels appear to gradually increase across EYO in both *APP* and *PSEN1* mutation carriers, despite only being significantly steeper in *PSEN1* mutation carriers compared to non-carriers. This plasma GFAP result differs from that already published by Johansson et al. (2022), likely due to the smaller sample size in the present study, thus, this result should be interpreted with care [13]. Nonetheless, the observed changes in these biomarkers suggest differing trajectories in *PSEN1* and *APP* mutation carriers.

Interestingly, an *AβPP*_arc_ mutation carrier had the highest plasma GFAP concentration (460.4 pg/ml), which does not appear to be because of human or experimental error. This individual did not show cerebral amyloid PET plaque deposition (Centiloid = −0.23), which was verified at autopsy at 66 years of age, where there was a lack of cored amyloid plaques observed by in vitro [3H] Pittsburgh Compound B binding and immunostaining [44]. Furthermore, high levels of plasma GFAP were also observed in other *APP* (ß_arc_) mutation carriers. Although it is well-documented in the literature that plasma GFAP is highly correlated with cerebral amyloid plaque deposition in vivo [37, 45–48], it is noteworthy that amyloid PET tracers have a low affinity to the neuropathologically distinct features of non-cored amyloid plaques observed in *AβPP*_arc_ mutation carriers. Our results suggest that plasma GFAP may not only be a proxy for cerebral amyloid plaque deposition, but also for soluble amyloid oligomers or atypical plaques that are often associated with specific ADAD mutations [49].

The significant association between increasing plasma GFAP concentration and decline in FDG PET uptake observed for *APP* mutation carriers compared to non-carriers suggests that pathological changes may be occurring earlier in these individuals compared to *PSEN1* mutation carriers. This is given that increasing plasma GFAP levels is an early AD phenomenon associated with increased cerebral amyloid production and deposition. This may be due to increased APP metabolism in *APP* mutation carriers resulting in an increase in amyloid isoforms, which may not be occurring in *PSEN1* mutation carriers. Interestingly, we found significant associations between subcortical FDG uptake and plasma GFAP in *APP* compared to *PSEN1* mutation carriers, but no significance with cortical regions. This suggests that decreasing glucose metabolism in subcortical brain regions is occurring earlier than changes in cortical regions with increasing plasma GFAP in the disease pathogenesis. Alternatively, the regional associations between FDG PET and plasma GFAP may reflect astrocyte heterogeneity across brain regions. Astrocytes differ in their molecular and functional profiles depending on brain regions, as shown in transcriptomic studies of AD brain tissue [50]. While the present study does not directly assess regional astrocyte activity in the brain, our group has previously reported regional differences in astrocyte activity using DED PET in ADAD and sporadic AD [12, 39], showing positive correlations between brain DED binding and FDG uptake, and negative correlations between brain DED binding and plasma GFAP. The latter is probably since plasma GFAP and brain DED binding represent markers of reactive astrogliosis but are detecting different states of astrogliosis subtypes [12].These findings suggest complex relationships between central and peripheral astrocyte markers, which may help explain the region-specific associations observed here. Nonetheless, plasma GFAP does not capture spatial information, and this represents a limitation when interpreting regional FDG changes in relation to peripheral astrocyte markers. Nonetheless, we provide evidence for different time courses and associations between biomarkers in different ADAD mutation groups, where *APP* mutation carriers may have pathophysiological trajectories more closely linked to amyloid production. Interestingly, within *PSEN1* mutations, it was recently shown that post-codon-200 mutation carriers showed a lower amyloid load in brain compared to pre-codon-200 carriers [51]. Lasty, we underline that there is a great requirement for new PET tracers for amyloid oligomers or atypical plaques, since plasma GFAP elevations seem to not be restricted to typical amyloid pathology.

A novelty to the present study is the investigation of the longitudinal associations between biomarkers over a large interval. We show that regional changes in FDG uptake are differentially associated with cognitive outcomes. We find that the typical temporoparietal region [7, 12, 52, 53] is significantly associated with a decline in all tests except RAVL Retention and Digit Span Forward in mutation carriers. However, going beyond this region, we found that a decline in cognitive outcomes was largely associated with decreasing FDG uptake in cortical regions, with more limited associations found with subcortical regions. Across all tested associations, FDG uptake was not significantly associated with Digit Span Forward, with limited associations with RAVL Retention. Digit Span Forward is one of the most preserved tests when assessing cognitive function and as a result it is often missed during a clinical assessment, therefore we did not expect a significant association between this test and FDG uptake. However, given RAVL Retention is a measure of memory, we expected a significant association between worsening scores and a decline in FDG uptake across the brain. We attribute this lack of significance to a large amount of missing data for this particular test in our sample and hypothesize that with a larger sample significance would emerge as is observed for the other tests. The lack of data for this test is likely due to clinical severity of the participants at the later time points and as such the test could not be performed. The differences in the significant associations between FDG uptake and cognitive decline were largely driven by *PSEN1* rather than *APP* carriers, providing further evidence for differing trajectories.

*APP* mutation carriers had a significant increase in plasma GFAP levels with declining glucose metabolism for most brain regions, and a steeper decline in all cognitive tests except for RAVL retention and Digit Span Forward. This may suggest that in these individuals, reactive astrocytes are consuming a large amount of brain-derived glucose [54], which is being reflected by the increasing plasma GFAP levels and decreasing FDG uptake, similar associations of which have been previously reported [11, 12, 30, 39]. This sequence of events is in line with other studies investigating plasma GFAP in clinical populations [55, 56]. This corroborates the positive association of astrocytic activity with glucose consumption in early stages of the continuum that fails at later stages of the disease, in which there is an inability to sustain the higher energy demands required by reactive astrocytes [30]. Overall, this improves our understanding of the preclinical phase of AD, supporting the notion that FDG PET is highly sensitive to astrocyte reactivity in AD, and emphasizes that astrocytes are a triggering factor of downstream neuropathological events in both ADAD and sporadic AD. This showcases the potential use of plasma GFAP as a useful biomarker for early (sporadic) AD detection, improving early diagnosis in the clinic. The lack of significance observed in *PSEN1* carriers may suggest some disease heterogeneity depending on the specific ADAD pathogenic variant, where *PSEN1* carriers have AD pathology closer to symptom onset, a phenomenon that has been previously hypothesized when investigating differences in, for example, white matter hyperintensities, amyloid deposition, and cognition [4, 44, 57–59].

One limitation of our study is the difference in follow-up time across mutations groups, with *AβPParc and PSEN1*_*M146V*_ carriers having a more limited follow-up interval. This limited follow-up could influence the estimated trajectories and reflect a potential survival or attrition bias. Despite these differences longitudinal analyses used linear mixed-effects models that accommodate unbalanced data. Furthermore, our study has a modest sample size, especially given specific pathogenic mutations result in differences within gene groups, e.g. in vitro and neuropathological differences between specific *APP* mutations. However, ADAD mutations in the population are extremely rare, therefore it is difficult to have large sample sizes. We compensate for this limitation by having a longitudinal study design with unique ADAD mutations that are not present in other ADAD cohorts, with the collection of three different modalities that coincide at multiple sampling points: PET, cognition, and plasma data. While results should be interpreted with care, they can be seen as hypothesis-generating, providing a platform for future research and validation. Furthermore, longitudinal studies in the AD field are still extremely lacking and our large time interval provides important insights into ADAD pathogenesis as the disease progresses from before to after symptom onset. Furthermore, it is one of the longest time intervals studied in the ADAD literature between different mutations, contrasting previously published ADAD results that analyse similar relationships but in a cross-sectional cohort without stratifying for specific mutations [11]. Although other plasma biomarkers such as NfL were not investigated in the present study, FDG-PET is considered to be a more established and sensitive marker of neurodegeneration. While plasma NfL correlates with FDG-PET [60], it is limited by age-related variability, methodological sensitivity, and lack of specificity to AD, with elevated levels also seen in other neurological conditions [61, 62]. Moreover, compared to other plasma biomarkers such as GFAP, NfL shows poorer discriminative performance for Aβ status [55] and demonstrates substantial variability among symptomatic carriers, with smaller differences between carriers and non-carriers, further reducing its utility in this context [13]. Many of the scans analysed were historical, which meant there was a limited scanner field of view, however no scans were removed from the analyses to retain the sample size. Therefore, we could not analyse changes in glucose metabolism in regions known to be vulnerable early in ADAD, such as the precuneus. However, the results we have presented corroborate published data, further cementing these ideas and hypotheses, and highlight the importance of having independent studies to confirm previously reported findings.

## Conclusion

To conclude, we show that there are longitudinal changes in AD biomarkers beginning approximately −20 to −10 years before symptom onset. Furthermore, we show associations in AD-related biomarkers in ADAD mutation carriers compared to non-carriers across EYO, with differences observed depending on *APP* or *PSEN1* mutation carriership. Specifically, we show that early changes in plasma GFAP are associated with early declines in FDG uptake and cognition only in *APP* carriers. This suggests *PSEN1* carriers have differing biomarker trajectories and biological mechanisms, which are changing later in the disease continuum, which may have implications in the clinic as well as when designing clinical interventions.

## Supplementary information

Supplementary Material

## Data Availability

Participants in this study belong to ADAD mutation families. To protect confidentiality of these individuals the data are not available to the public since anonymization cannot be guaranteed due to the small sample size. Anonymized data will be shared by request from a qualified academic investigator for replication of procedures and results presented in the article and if data transfer is in agreement with EU legislation on the general data protection regulation and decisions by the Ethical Review Board of Sweden. Sharing of data should be regulated in a material transfer agreement and or data processing agreement as appropriate.
