# Supplementary material for: Early functional changes and plasma GFAP in Swedish families with Autosomal Dominant Alzheimer’s disease mutations
Source: Transl Psychiatry. 2026 Jan 27;16:67. doi: 10.1038/s41398-026-03829-6 (PMC12877000; doi:10.1038/s41398-026-03829-6)
Supplement: Supplementary file 1 — Supplementary Material [file 41398_2026_3829_MOESM1_ESM.pdf]

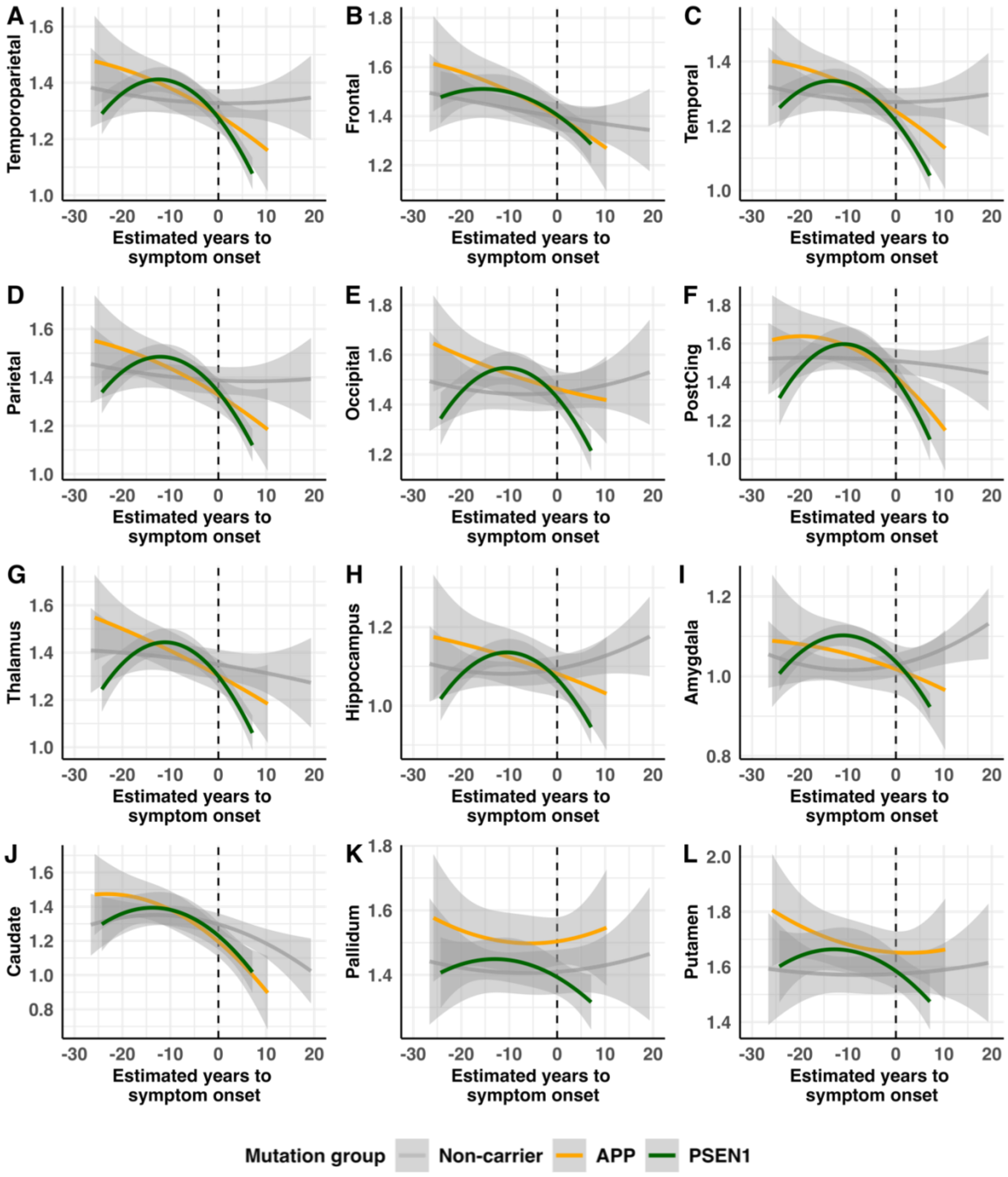

Supplementary Figure 1 Longitudinal trajectories of FDG SUVRs in ADAD mutation carriers and non-carriers with respect to symptom onset.

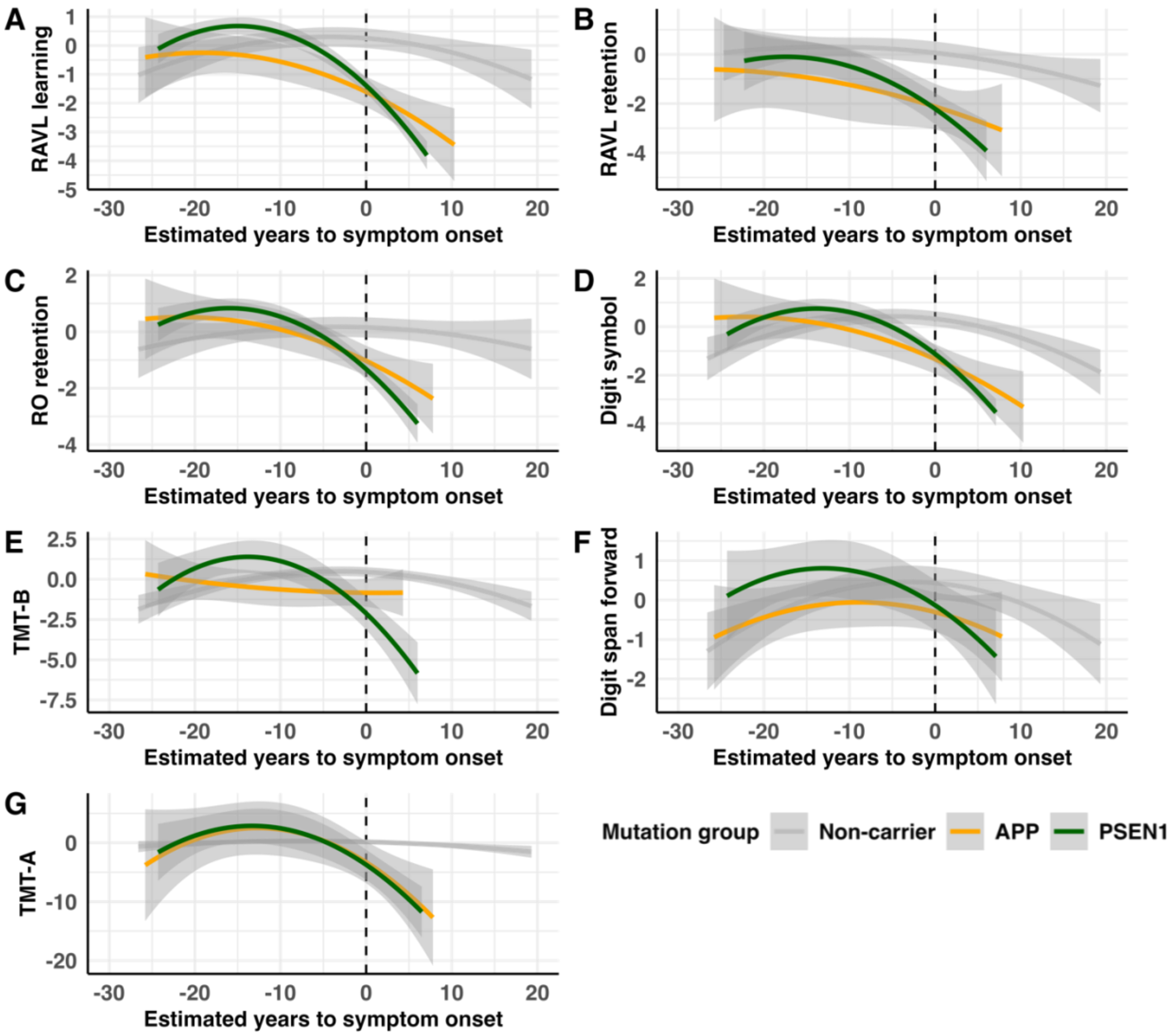

Supplementary Figure 2 Longitudinal trajectories of cognitive tests in ADAD mutation carriers and non-carriers.

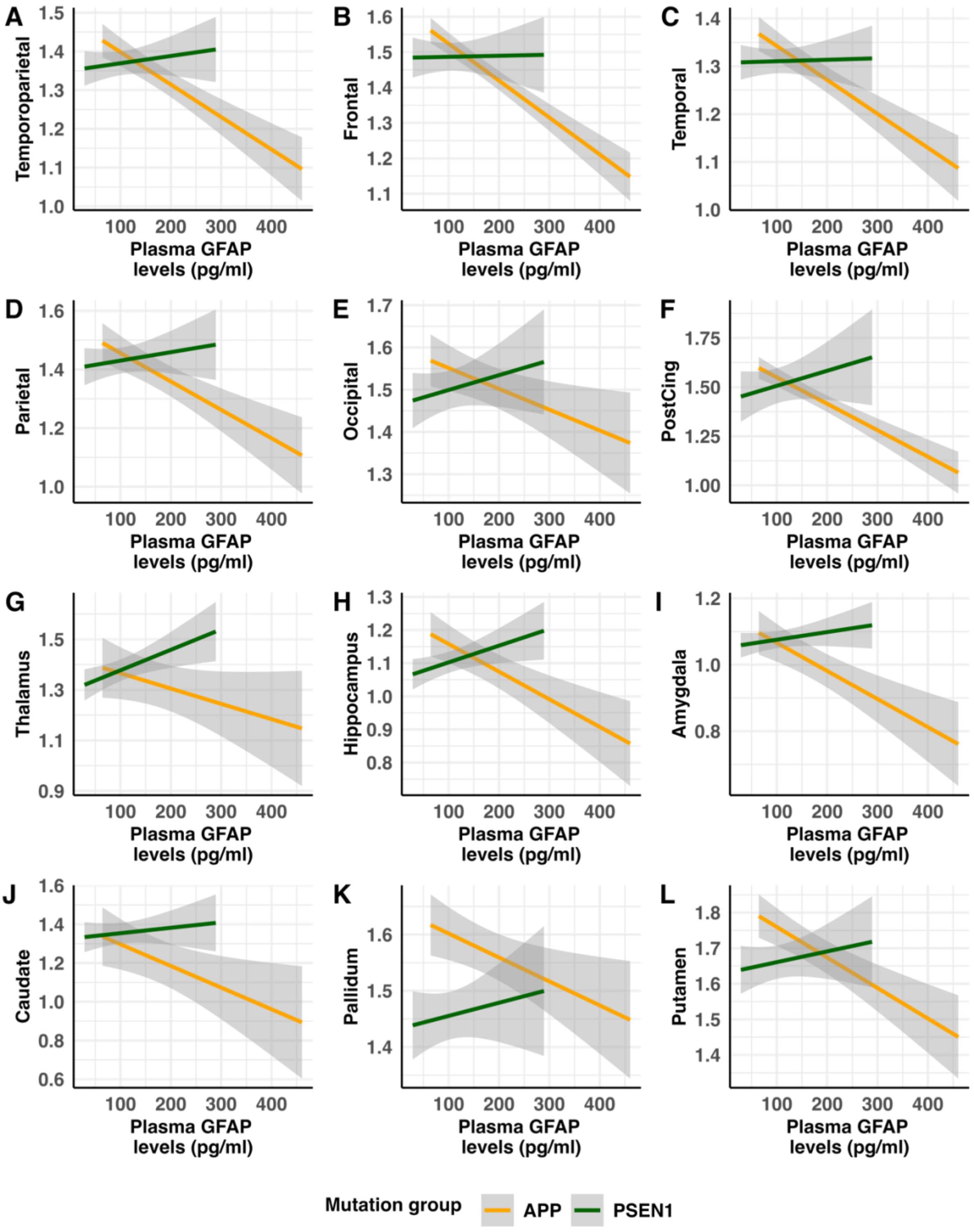

Supplementary Figure 3 Longitudinal relationships between FDG SUVRs and plasma GFAP in *APP* and *PSEN1* mutation carriers.

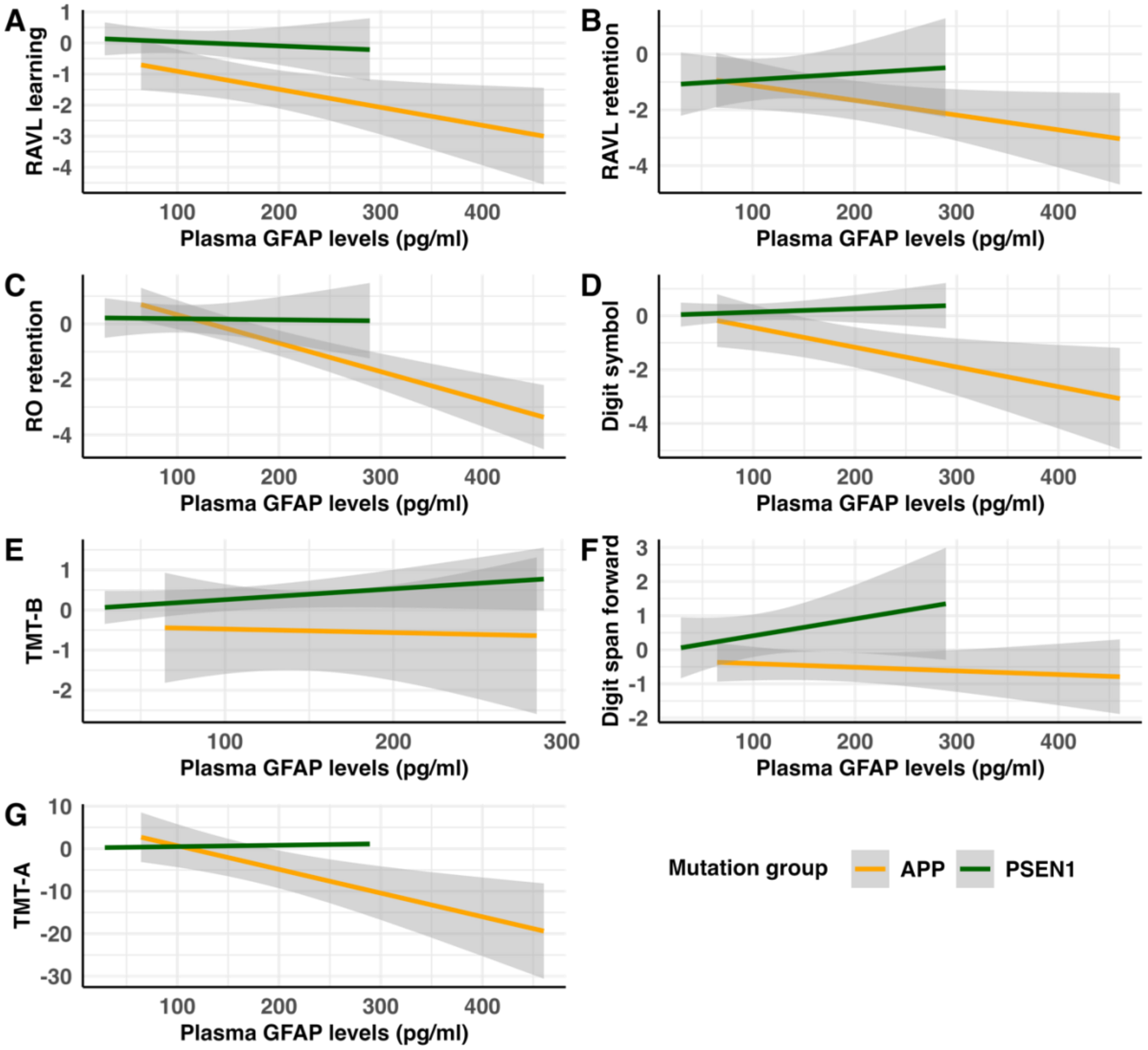

Supplementary Figure 4 Longitudinal relationships between plasma GFAP and cognition in *PSEN1* and *APP* mutation carriers and non-carriers.

Supplementary Table 1 Longitudinal trajectories of [18F]FDG PET across estimated year to symptom onset in ADAD mutation carriers compared to non-carries.

	EYO*noMC-MC			EYO*-noMC-*PSEN1*			EYO*noMC-*APP*			EYO*APP-*PSEN1*		
**Outcome**	**Estimate (C.I.)**	***t*-value**	***P*-value**	**Estimate (C.I.)**	***t*-value**	***P*-value**	**Estimate (C.I.)**	***t*-value**	***P*-value**	**Estimate (C.I.)**	***t*-value**	***P*-value**
**Frontal**	-6.05e-03 (-0.01, -0.0003)	-2.08	**0.041**	-6.61e-03 (-0.01, 0.0003)	-1.87	0.065	-5.79e-03 (-0.01, 0.001)	-1.65	0.103	-1.04e-03 (-0.01, 0.01)	-0.31	0.755
**Temporal**	-6.38e-03 (-0.01, -0.002)	-2.88	**0.005**	7.334-03 (-0.01, -0.002)	-2.74	**0.007**	-6.29e-03 (-0.01, -0.001)	-2.30	**0.024**	-2.93e-03 (-0.01, 0.001)	-1.30	0.201
**Parietal**	-8.13e-03 (-0.01, -0.002)	-2.73	**0.008**	-8.509e-03 (-0.02, -0.001)	-2.36	**0.021**	-8.40e-03 (-0.02, -0.001)	-2.28	**0.025**	-8.04e-04 (-0.01, 0.01)	-0.26	0.796
**Occipital**	-3.72e-03 (-0.01, 0.003)	-1.05	0.297	-3.88e-03 (-0.01, 0.004)	-0.91	0.363	-4.80e-03 (-0.01, 0.004)	-1.10	0.273	-2.50e-03 (-0.01, 0.005)	-0.67	0.510
**Posterior cingulate**	-1.08e-02 (-0.02, -0.003)	-2.89	**0.005**	-9.50e-03 (-0.02, -0.001)	-2.11	**0.038**	-1.32e-02 (-0.02, -0.004)	-2.85	**0.005**	9.69e-04 (-0.01, 0.01)	0.22	0.830
**Temporoparietal**	-7.30e-03 (-0.01, -0.002)	-2.82	**0.006**	-7.86e-03 (-0.01, -0.002)	-2.52	**0.014**	-7.44e-03 (-0.01, -0.001)	-2.33	**0.022**	-1.89e-03 (-0.01, 0.003)	-0.72	0.476
**Thalamus**	-6.43e-03 (-0.01, -0.0002)	-2.03	**0.045**	-3.84e-03 (-0.01, 0.004)	-1.013	0.314	-9.88e-03 (-0.02, -0.002)	-2.55	**0.013**	0.0047305 (-0.003, 0.01)	1.26	0.217
**Hippocampus**	-2.14e-03 (-0.01, 0.002)	-1.15	0.254	-2.15e-03 (-0.01, 0.002)	-0.967	0.337	-2.69e-03 (-0.01, 0.002)	-1.18	0.242	-3.89e-04 (-0.01, 0.001)	-1.52	0.135
**Amygdala**	-3.17e-03 (-0.01, 0.0004)	-1.75	0.084	-2.78e-03 (-0.01, 0.002)	-1.262	0.210	-3.69e-03 (-0.01, 0.001)	-1.64	0.105	-3.04e-03 (-0.01, 0.002)	-1.28	0.208
**Caudate**	-1.14e-02 (-0.02, -0.005)	-3.31	**0.001**	-9.57e-03 (-0.02, -0.001)	-2.312	**0.023**	-1.36e-02 (-0.02, -0.01)	-3.22	**0.002**	0.0021365 (-0.01, 0.01)	0.44	0.666
**Pallidum**	-1.45e-03 (-0.01, 0.005)	-0.45	0.651	-3.38e-03 (-0.01, 0.004)	-0.885	0.378	-3.99e-04 (-0.01, 0.01)	-0.10	0.919	-4.71e-03 (-0.01, 0.003)	-1.27	0.213
**Putamen**	-1.94e-03 (-0.01, 0.005)	-0.56	0.579	-2.45e-03 (-0.01, 0.01)	-0.582	0.562	-2.32e-03 (-0.01, 0.01)	-0.54	0.591	-3.64e-03 (-0.01, 0.005)	-0.85	0.400

Abbreviations: CI = 95% confidence interval; EYO = estimated years to symptom onset; MC = ADAD mutation carrier; noMC = ADAD mutation non-carrier

Supplementary Table 2 Longitudinal trajectories of cognitive test scores across estimated year to symptom onset in ADAD mutation carriers compared to non-carries.

Outcome	EYO*noMC-MC			EYO*noMC-*PSEN1*			EYO*noMC-*APP*			EYO*APP-*PSEN1*		
	Estimate (C.I.)	*t*-value	*P*-value	Estimate (C.I.)	*t*-value	*P*-value	Estimate (C.I.)	*t*-value	*P*-value	Estimate (C.I.)	*t*-value	*P*-value
**RAVL learning**	-0.14 (-0.16, -0.09)	-6.37	**1.28e-08**	-0.15 (-0.20, -0.10)	-6.27	**1.83e-08**	-9.52e-02 (-0.14, -0.05)	-3.94	**1.66 e-4**	-0.04 (-0.08, 0.0002)	-1.95	0.058
**RAVL retention**	-0.09 (-0.15, -0.04)	-3.23	**0.002**	-0.12 (-0.19, -0.04)	-3.18	**0.002**	-0.07 (-0.13, -0.001)	-1.99	0.052	-0.03 (-0.12, 0.06)	-0.67	0.512
**RO retention**	-0.12 (-0.16, -0.08)	-6.28	**9.14e-09**	-0.13 (-0.17, -0.08)	-5.29	**7.85e-07**	-0.12 (-0.17, -0.07)	-5.01	**2.43e-06**	-0.05 (-0.09, 0.001)	-1.92	0.063
**Digit Symbol**	-0.12 (-0.15, -0.08)	-6.78	**1.07e-09**	-0.14 (-0.18, -0.10)	-6.94	**4.26e-10**	-9.31e-02 (-0.13, -0.05)	-4.53	**1.69e-05**	-0.09 (-0.14, -0.05)	-3.812	**4.31e-04**
**TMT-B**	-0.16 (-0.22, -0.10)	5.39	**7.71e-07**	-0.21 (-0.28, -0.14)	-6.17	**7.35e-08**	-0.118 (-0.18, -0.04)	-2.92	**0.005**	-0.11 (-0.21, -0.01)	-2.22	**0.032**
**Digit Span Forward**	-0.05 (-0.09, -0.01)	-2.64	**0.011**	-0.07 (-0.12, -0.02)	-2.80	**0.006**	-0.04 (-0.08, 0.01)	-1.45	0.151	-0.05 (-0.11, 0.01)	-1.54	0.132
**TMT-A**	-0.40 (-0.55, -0.24)	-5.05	**1.99e-06**	-0.42 (-0.60, -0.25)	-4.65	**1.00e-05**	-0.36 (-0.57, -0.15)	-3.40	**9.59e-4**	-0.08 (-0.37, 0.22)	-0.49	0.627

Abbreviations: CI = 95% confidence interval; EYO = estimated years to symptom onset; MC = ADAD mutation carrier; noMC = ADAD mutation non-carrier : RAVL = Rey Auditory Verbal Learning; RO = Rey-Osterrieth Complex Figure; TMT = Trail Making Test

Supplementary Table 3 Longitudinal associations between cortical [18F]FDG PET and cognitive outcomes in ADAD mutation carriers compared to non-carries.

Outcome	Temporoparietal*noMC-MC			Frontal*noMC-MC			Temporal*noMC-MC			Parietal*noMC-MC			Occipital*noMC-MC			Posterior cingulate*noMC-MC		
	Estimate (C.I.)	*t*-value	*P*-value	Estimate (C.I.)	*t*-value	*P*-value	Estimate (C.I.)	*t*-value	*P*-value	Estimate (C.I.)	*t*-value	*P*-value	Estimate (C.I.)	*t*-value	*P*-value	Estimate (C.I.)	*t*-value	*P*-value
**RAVL learning**	4.47 (1.43, 7.52)	2.78	**0.005**	3.78 (1.10, 6.45)	2.77	**0.007**	5.60 (2.03, 9.16)	3.08	**0.003**	3.555 (0.93, 6.18)	2.66	**0.009**	0.902 (-1.73, 3.54)	0.67	0.503	1.927 (-0.10, 3.95)	1.86	0.065
**RAVL retention**	4.105 (0.10, 8.69)	2.01	0.050	4.54 (1.13, 7.96)	2.61	**0.011**	5.45 (0.31, 10.58)	2.08	**0.042**	3.450 (-0.12, 7.02)	1.89	0.063	-0.181 (-4.41, 4.04)	-0.08	0.933	1.515 (-1.47, 4.50)	0.99	0.324
**RO retention**	5.18 (1.98, 8.39)	3.17	**0.002**	5.33 (2.79, 7.87)	4.11	**8.49e-05**	5.92 (2.09, 9.75)	3.03	**0.003**	4.353 (1.68, 7.02)	3.19	**0.002**	0.064 (-2.68, 2.81)	0.05	0.963	3.611 (1.75, 5.47)	3.80	**2.67e-04**
**Digit Symbol**	5.79 (3.27, 8.31)	4.51	**1.79e-05**	3.60 (1.30, 5.90)	3.07	**0.003**	6.96 (4.04, 9.88)	4.67	**8.99e-06**	4.749 (2.58, 6.92)	4.29	**4.08e-05**	3.107 (0.87, 5.34)	2.72	**0.007**	2.795 (1.08, 4.51)	3.20	**0.002**
**TMT-B**	11.83 (6.74, 16.93)	4.55	**1.67e-05**	9.79 (5.96, 13.63)	5.01	**3.38e-08**	13.67 (7.64, 19.69)	4.45	**2.68e-05**	9.678 (5.36, 13.99)	4.40	**2.97e-05**	2.466 (-2.16, 7.10)	1.04	0.299	5.143 (2.11, 8.18)	3.32	**0.001**
**Digit Span Forward**	0.65 (-2.37, 3.66)	0.42	0.676	0.89 (-1.67, 3.45)	0.68	0.499	1.17 (-2.37, 4.71)	0.65	0.518	0.351 (-2.22, 2.92)	0.27	0.789	-0.476 (-3.03, 2.08)	-0.37	0.716	0.622 (-1.30, 2.55)	0.63	0.528
**TMT-A**	32.76 (22.27, 43.25)	6.12	**1.78e-08**	25.20 (14.24, 36.15)	4.51	**1.98e-05**	36.39 (21.78, 51.01)	4.88	**5.40e-06**	29.402 (20.37, 38.44)	6.38	**5.45e-09**	21.585 (11.80, 31.37)	4.32	**3.63e-05**	17.303 (9.84, 24.77)	4.54	**4.54e-05**

Abbreviations: CI = 95% confidence interval; EYO = estimated years to symptom onset; MC = ADAD mutation carrier; noMC = ADAD mutation non-carrier

Supplementary Table 4 Longitudinal associations between subcortical [18F]FDG PET and cognitive outcomes in ADAD mutation carriers compared to non-carries.

	Thalamus*noMC-MC			Hippocampus*noMC-MC			Amygdala*noMC-MC			Caudate*noMC-MC			Pallidum*noMC-MC			Putamen*noMC-MC		
Outcome	Estimate (C.I.)	*t*-value	*P*-value	Estimate (C.I.)	*t*-value	*P*-value	Estimate (C.I.)	*t*-value	*P*-value	Estimate (C.I.)	*t*-value	*P*-value	Estimate (C.I.)	*t*-value	*P*-value	Estimate (C.I.)	*t*-value	*P*-value
**RAVL learning**	3.08 (0.45, 5.70)	2.30	**0.024**	0.91 (-4.15, 5.98)	0.35	0.725	3.98 (-1.42, 9.39)	1.44	0.152	2.85 (0.86, 4.84)	2.80	**0.006**	0.59 (-2.57, 3.76)	0.37	0.714	-0.86 (-3.68, 1.95)	-0.60	0.549
**RAVL retention**	2.45 (-1.07, 5.98)	1.363	0.1785	2.34 (-4.57, 9.25)	0.66	0.511	4.87 (-2.72, 12.46)	1.26	0.213	1.58 (-1.10, 4.26)	1.15	0.253	1.58 (-2.74, 5.90)	0.72	0.477	-0.11 (-4.42, 4.20)	-0.05	0.960
**RO retention**	5.09 (2.58, 7.60)	3.97	**1.4e-04**	2.69 (-2.39, 7.76)	1.04	0.302	4.66 (-0.80, 10.13)	1.67	0.098	5.04 (3.18, 6.90)	5.31	**7.47e-07**	1.71 (-1.28, 4.69)	1.12	0.265	1.58 (-1.52, 4.67)	0.99	0.321
**Digit Symbol**	3.94 (1.78, 6.11)	3.56	**5.54e-04**	1.38 (-3.00, 5.75)	0.62	0.538	5.50 (1.16, 9.84)	2.49	**0.014**	3.45 (1.78, 5.12)	4.05	**1.02e-04**	1.29 (-1.38, 3.97)	0.95	0.346	1.38 (-1.20, 3.96)	1.05	0.296
**TMT-B**	5.31 (1.29, 9.32)	2.59	**0.011**	1.76 (-6.10, 9.62)	0.44	0.662	6.39 (-2.32, 15.09)	1.44	0.154	6.86 (4.42, 9.30)	5.51	**1.63e-06**	2.95 (-1.76, 7.66)	1.23	0.223	3.05 (-1.61, 7.70)	1.28	0.204
**Digit Span Forward**	0.89 (-1.60, 3.38)	0.70	0.485	-0.96 (-5.57, 3.64)	-0.41	0.682	1.09 (-4.06, 6.25)	0.42	0.679	0.90 (-1.08, 2.88)	0.89	0.375	-1.36 (-4.25, 1.53)	-0.92	0.358	0.70 (-2.08, 3.47)	0.49	0.623
**TMT-A**	20.73 (9.77, 31.69)	3.71	**3.77e-04**	29.90 (12.74, 47.07)	3.41	**1.39e-03**	46.46 (26.41, 66.50)	4.54	**1.38e-05**	18.59 (9.76, 27.43)	4.13	**7.70-05**	12.97 (-0.18, 26.12)	1.93	0.058	22.08 (10.58, 33.57)	3.76	**9.85e-04**

Abbreviations: CI = 95% confidence interval; EYO = estimated years to symptom onset; MC = ADAD mutation carrier; noMC = ADAD mutation non-carrier

Supplementary Table 5 Longitudinal associations between cortical [18F]FDG PET and cognitive test scores across estimated year to symptom onset in *APP* mutation carriers compared to non-carries.

	Temporoparietal*noMC-*APP*			Frontal*noMC-*APP*			Temporal*noMC-*APP*			Parietal*noMC-*APP*			Occipital*noMC-*APP*			Posterior cingulate*noMC-*APP*		
Outcome	Estimate (C.I.)	*t*-value	*P*-value	Estimate (C.I.)	*t*-value	*P*-value	Estimate (C.I.)	*t*-value	*P*-value	Estimate (C.I.)	*t*-value	*P*-value	Estimate (C.I.)	*t*-value	*P*-value	Estimate (C.I.)	*t*-value	*P*-value
**RAVL learning**	4.55 (0.75, 8.35)	2.35	**0.021**	3.44 (0.06, 6.82)	2.00	**0.049**	5.53 (1.16, 9.91)	2.48	**0.015**	3.58 (0.28, 6.89)	2.13	**0.037**	2.34 (-1.32, 6.00)	1.25	0.213	2.58 (-0.09, 5.25)	1.89	0.062
**RAVL retention**	2.86 (-1.99, 7.70)	1.16	0.252	3.07 (-1.13, 7.27)	1.43	0.156	3.67 (-2.18, 9.51)	1.23	0.223	2.24 (-1.80, 6.27)	1.09	0.282	0.56 (-4.82, 5.93)	0.20	0.840	0.63 (-2.98, 4.23)	0.34	0.734
**RO retention**	3.27 (-0.57, 7.11)	1.67	0.098	3.28 (0.31, 6.26)	2.16	**0.033**	3.88 (-0.55, 8.32)	1.72	0.090	2.75 (-0.57, 6.07)	1.63	0.107	0.13 (-3.25, 3.51)	0.08	0.940	3.21 (0.60, 5.82)	2.41	**0.018**
**Digit Symbol**	3.28 (0.06, 6.49)	2.00	**0.048**	1.56 (-1.25, 4.37)	1.09	0.278	3.34 (-0.29, 6.96)	1.81	0.074	3.12 (0.29, 5.94)	2.16	**0.033**	1.49 (-1.50, 4.49)	0.98	0.331	2.83 (0.45, 5.21)	2.33	**0.022**
**TMT-B**	2.97 (-3.52, 9.45)	0.90	0.373	2.01 (-2.26, 6.29)	0.92	0.358	2.88 (-4.24, 10.00)	0.79	0.430	2.99 (-2.89, 8.86)	1.00	0.322	0.80 (-5.49, 7.09)	0.25	0.804	1.37 (-3.30, 6.03)	0.58	0.567
**Digit Span Forward**	-0.88 (-4.33, 2.57)	-0.50	0.617	-0.75 (-3.56, 2.06)	-0.52	0.603	-0.90 (-4.83, 3.04)	-0.45	0.656	-0.73 (-3.74, 2.29)	-0.47	0.639	-0.86 (-3.95, 2.22)	-0.55	0.584	0.07 (-2.39, 2.53)	0.06	0.956
**TMT-A**	25.23 (12.16, 38.29)	3.79	**0.0002**	17.59 (4.54, 30.64)	2.64	**0.009**	18.01 (0.46, 35.56)	2.011	**0.047**	24.29 (13.07, 35.51)	4.24	**4.90e-05**	13.56 (0.37, 26.74)	2.02	**0.047**	18.21 (8.31, 28.10)	3.61	**0.0005**

Abbreviations: CI = 95% confidence interval; EYO = estimated years to symptom onset; MC = ADAD mutation carrier; noMC = ADAD mutation non-carrier : RAVL = Rey Auditory Verbal Learning; RO = Rey-Osterrieth Complex Figure; TMT = Trail Making Test

Supplementary Table 6 Longitudinal associations between subcortical [18F]FDG PET and cognitive test scores across estimated year to symptom onset in *APP* mutation carriers compared to non-carries.

Outcome	Thalamus *noMC-*APP* Estimate (C.I.)	t-value	P-value	Hippocampus *noMC-*APP* Estimate (C.I.)	t-value	P-value	Amygdala *noMC-*APP* Estimate (C.I.)	t-value	P-value	Caudate *noMC-*APP* Estimate (C.I.)	t-value	P-value	Pallidum *noMC-*APP* Estimate (C.I.)	t-value	P-value	Putamen *noMC-*APP* Estimate (C.I.)	t-value	P-value
**RAVL learning**	2.83 (-0.50, 6.15)	1.67	0.099	1.87 (-4.12, 7.85)	0.61	0.542	2.19 (-3.90, 8.28)	0.70	0.483	2.44 (-0.01, 4.89)	1.96	**0.054**	2.04 (-2.03, 6.10)	0.98	0.328	1.22 (-2.84, 5.29)	0.59	0.557
**RAVL retention**	0.23 (-3.99, 4.45)	0.11	0.915	1.74 (-5.75, 9.23)	0.45	0.651	4.18 (-4.01, 12.37)	0.99	0.321	0.10 (-3.19, 3.40)	0.06	0.951	2.69 (-2.94, 8.32)	0.94	0.354	0.12 (-5.49, 5.73)	0.04	0.966
**RO retention**	3.47 (0.58, 6.35)	2.35	**0.021**	2.51 (-3.19, 8.21)	0.86	0.390	5.13 (-1.26, 11.51)	1.57	0.119	3.87 (1.58, 6.17)	3.30	**0.001**	1.38 (-2.13, 4.88)	0.77	0.443	2.39 (-1.42, 6.20)	1.23	0.222
**Digit Symbol**	1.22 (-1.38, 3.82)	0.92	0.360	0.42 (-4.75, 5.59)	0.16	0.873	2.14 (-2.95, 7.23)	0.82	0.412	1.58 (-0.39, 3.55)	1.57	**0.119**	-0.12 (-3.43, 3.18)	-0.07	0.941	0.82 (-2.67, 4.32)	0.46	0.646
**TMT-B**	0.98 (-3.75, 5.70)	0.40	0.687	-3.55 (-12.99, 5.89)	-0.74	0.464	-1.04 (-10.23, 8.16)	-0.22	0.825	1.71 (-1.08, 4.50)	1.20	0.232	1.49 (-4.43, 7.42)	0.49	0.622	0.78 (-5.29, 6.85)	0.25	0.801
**Digit Span Forward**	0.57 (-2.45, 3.59)	0.37	0.710	-2.20 (-7.28, 2.88)	-0.85	0.399	-0.28 (-6.04, 5.48)	-0.10	0.924	-0.22 (-2.58, 2.14)	-0.18	0.856	-2.30 (-5.70, 1.10)	-1.33	0.188	0.23 (-3.03, 3.50)	0.14	0.888
**TMT-A**	5.01 (-7.77, 17.80)	0.77	0.444	32.36 (11.58, 53.14)	3.05	**0.003**	37.34 (15.38, 59.31)	3.33	**0.003**	10.45 (-0.48, 21.37)	1.87	0.064	1.66 (-14.30, 17.61)	0.20	**0.839**	16.79 (2.20, 31.37)	2.26	0.089

Abbreviations: CI = 95% confidence interval; EYO = estimated years to symptom onset; MC = ADAD mutation carrier; noMC = ADAD mutation non-carrier : RAVL = Rey Auditory Verbal Learning; RO = Rey-Osterrieth Complex Figure; TMT = Trail Making Test

Supplementary Table 7 Longitudinal associations between cortical [18F]FDG PET and cognitive test scores across estimated year to symptom onset in *PSEN1* mutation carriers compared to non-carries

Outcome	Temporoparietal*noMC-*PSEN1* Estimate (C.I.)	t-value	P-value	Frontal*noMC-*PSEN1* Estimate (C.I.)	t-value	P-value	Temporal*noMC-*PSEN1* Estimate (C.I.)	t-value	P-value	Parietal*noMC-*PSEN1* Estimate (C.I.)	t-value	P-value	Occipital*noMC-*PSEN1* Estimate (C.I.)	t-value	P-value	Posterior cingulate*noMC-*PSEN1* Estimate (C.I.)	t-value	P-value
**RAVL learning**	5.00 (1.37, 8.62)	2.70	**0.008**	4.01 (0.80, 7.22)	2.45	**0.016**	6.60 (2.35, 10.85)	3.04	**0.003**	3.77 (0.65, 6.90)	2.37	**0.020**	0.73 (-2.46, 3.92)	0.45	0.653	1.87 (-0.32, 4.05)	1.68	0.097
**RAVL retention**	7.14 (1.71, 12.56)	2.58	**0.012**	5.72 (1.75, 9.69)	2.83	**0.006**	7.81 (1.36, 14.27)	2.37	**0.021**	6.09 (1.51, 10.67)	2.61	**0.011**	-1.61 (-7.29, 4.06)	-0.56	0.580	2.25 (-1.11, 5.61)	1.31	0.195
**RO retention**	7.45 (3.25, 11.65)	3.48	**0.0008**	7.68 (4.49, 10.88)	4.72	**1.19e-05**	9.07 (3.73, 14.41)	3.33	**0.001**	5.85 (2.51, 9.18)	3.44	**0.001**	0.2 (-3.64, 4.15)	0.13	0.899	3.80 (1.80, 5.79)	3.73	**3.67e-04**
**Digit Symbol**	7.61 (4.69, 10.53)	5.11	**1.78e-06**	5.58 (2.90, 8.27)	4.08	**0.0001**	9.80 (6.43, 13.16)	5.70	**1.44e-07**	5.88 (3.36, 8.40)	4.57	**1.60e-05**	4.434 (1.67, 7.20)	3.14	**0.002**	2.78 (0.93, 4.63)	2.94	**0.004**
**TMT-B**	20.54 (14.55, 26.53)	6.72	**1.99e-09**	17.95 (13.82, 22.09)	8.51	**3.05e-11**	26.61 (19.43, 33.79)	7.27	**2.11e-10**	15.21 (10.09, 20.34)	5.82	**8.15e-08**	5.624 (-1.15, 12.40)	1.63	0.107	6.94 (3.69, 10.20)	4.19	**6.37e-05**
**Digit Span Forward**	2.78 (-1.03, 6.59)	1.43	0.156	3.50 (0.14, 6.86)	2.04	**0.044**	4.57 (0.06, 9.08)	1.99	**0.05**	1.60 (-1.62, 4.81)	0.97	0.333	0.93 (-2.63, 4.48)	0.51	0.611	0.96 (-1.12, 3.04)	0.91	0.368
**TMT-A**	42.08 (28.95, 55.22)	6.28	**8.67e-09**	35.52 (21.01, 50.03)	4.80	**5.94e-06**	60.35 (42.69, 78.00)	6.70	**1.76e-09**	35.66 (24.05, 47.27)	6.02	**2.84e-08**	32.60 (19.74, 45.46)	4.97	**2.77e-06**	17.56 (8.78, 26.34)	3.92	**1.61e-04**

Abbreviations: CI = 95% confidence interval; EYO = estimated years to symptom onset; MC = ADAD mutation carrier; noMC = ADAD mutation non-carrier : RAVL = Rey Auditory Verbal Learning; RO = Rey-Osterrieth Complex Figure; TMT = Trail Making Test

Supplementary Table 8 Longitudinal associations between subcortical [18F]FDG PET and cognitive test scores across estimated year to symptom onset in *PSEN1* mutation carriers compared to non-carries

Outcome	Thalamus *noMC-*PSEN1*			Hippocampus *noMC-*PSEN1*			Amygdala *noMC-*PSEN1*			Caudate*noMC-*PSEN1*			Pallidum *noMC-*PSEN1*			Putamen *noMC-*PSEN1*		
	Estimate (C.I.)	*t*-value	*P*-value	Estimate (C.I.)	*t*-value	*P*-value	Estimate (C.I.)	*t*-value	*P*-value	Estimate (C.I.)	*t*-value	*P*-value	Estimate (C.I.)	*t*-value	*P*-value	Estimate (C.I.)	*t*-value	*P*-value
**RAVL learning**	3.77 (0.50, 7.05)	2.26	**0.026**	1.15 (-5.34, 7.64)	0.35	0.729	7.16 (0.03, 14.29)	1.97	**0.052**	3.10 (0.76, 5.43)	2.60	**0.011**	-0.07 (-4.43, 4.29)	-0.03	0.975	-1.73 (-5.08, 1.63)	-1.01	0.316
**RAVL retention**	5.46 (1.14, 9.78)	2.48	**0.033**	7.51 (-0.54, 15.56)	1.83	0.072	11.46 (1.54, 21.38)	2.26	**0.027**	2.58 (-0.46, 5.62)	1.67	0.102	2.95 (-3.19, 9.09)	0.94	0.350	0.72 (-4.89, 6.32)	0.25	0.803
**RO retention**	7.95 (4.32, 11.58)	4.29	**4.70e-05**	3.29 (-4.06, 10.64)	0.88	0.382	3.39 (-4.26, 11.04)	0.87	**0.388**	5.97 (3.80, 8.14)	5.39	**6.80e-07**	3.16 (-1.61, 7.94)	1.30	0.198	0.90 (-3.75, 5.55)	0.38	0.704
**Digit Symbol**	6.46 (3.86, 9.07)	4.86	**4.83e-06**	2.61 (-3.09, 8.32)	0.90	0.372	9.37 (4.01, 14.73)	3.42	**0.001**	5.12 (3.32, 6.92)	5.58	**3.19e-07**	3.23 (-0.52, 6.98)	1.69	0.094	1.90 (-1.45, 5.25)	1.11	0.270
**TMT-B**	11.95 (6.44, 17.47)	4.25	**6.86e-05**	11.05 (1.05, 21.05)	2.16	**0.035**	21.55 (9.94, 33.15)	3.64	**5.34e-04**	12.66 (9.99, 15.33)	9.29	**1.76e-14**	8.16 (0.41, 15.92)	2.06	**0.042**	7.86 (1.29, 14.43)	2.34	**0.023**
**Digit Span Forward**	0.57 (-2.45, 3.59)	0.92	0.361	2.34 (-3.73, 8.42)	0.76	0.452	2.94 (-3.96, 9.84)	0.84	0.406	1.87 (-0.44, 4.18)	1.58	0.117	2.11 (-2.08, 6.30)	0.99	0.327	3.64 (-0.21, 7.50)	1.85	0.069
**TMT-A**	43.54 (30.03, 57.04)	6.32	**8.09e-09**	31.81 (9.84, 53.78)	2.84	**0.005**	59.30 (34.22, 84.39)	4.63	**1.61e-04**	26.11 (15.85, 36.37)	4.99	**2.80e-06**	37.30 (17.43, 57.16)	3.68	**4.24e-04**	31.62 (16.83, 46.40)	4.19	**0.167**

Abbreviations: CI = 95% confidence interval; EYO = estimated years to symptom onset; MC = ADAD mutation carrier; noMC = ADAD mutation non-carrier : RAVL = Rey Auditory Verbal Learning; RO = Rey-Osterrieth Complex Figure; TMT = Trail Making Test

Supplementary Table 9 Longitudinal associations between cortical [18F]FDG PET and cognitive test scores across estimated year to symptom onset in *APP* carriers compared to *PSEN1* carriers

Outcome	Temporoparietal**PSEN1-APP*			Frontal**PSEN1-APP*			Temporal**PSEN1-APP*			Parietal**PSEN1-APP*			Occipital**PSEN1-APP*			Posterior cingulate**PSEN1-APP*		
	Estimate (C.I.)	*t*-value	*P*-value	Estimate (C.I.)	*t*-value	*P*-value	Estimate (C.I.)	*t*-value	*P*-value	Estimate (C.I.)	*t*-value	*P*-value	Estimate (C.I.)	*t*-value	*P*-value	Estimate (C.I.)	*t*-value	*P*-value
**RAVL learning**	-0.08 (-3.22, 3.07)	-0.05	0.963	-0.12 (-2.95, 2.71)	-0.09	0.932	-0.38 (-4.02, 3.27)	-0.20	0.840	0.23 (-2.54, 3.00)	0.16	0.873	-1.38 (-4.56, 1.80)	-0.85	0.399	0.01 (-1.91, 1.94)	0.01	0.989
**RAVL retention**	-0.56 (-7.42, 6.29)	-0.16	0.875	0.68 (-4.02, 5.37)	0.28	0.779	1.06 (-6.25, 8.36)	0.28	0.779	-0.69 (-6.62, 5.25)	-0.23	0.823	-5.06 (-12.25, 2.14)	-1.38	0.180	0.44 (-3.64, 4.52)	0.21	0.835
**RO retention**	2.79 (-1.35, 6.92)	1.32	0.194	2.80 (-0.37, 5.98)	1.73	0.092	2.31 (-2.78, 7.41)	0.89	0.378	2.68 (-0.67, 6.04)	1.57	0.125	-1.16 (-4.90, 2.59)	-0.61	0.549	1.59 (-0.49, 3.67)	1.50	0.143
**Digit Symbol**	2.76 (-0.86, 6.37)	1.49	0.142	1.79 (-1.57, 5.14)	1.04	0.302	4.84 (0.77, 8.91)	2.33	**0.024**	1.42 (-1.76, 4.61)	0.87	0.386	0.97 (-2.43, 4.37)	0.56	0.578	-0.12 (-2.44, 2.19)	-0.10	0.918
**TMT-B**	16.11 (6.39, 25.83)	3.25	**0.002**	13.43 (7.54, 19.32)	4.47	**6.9e-05**	19.81 (8.79, 30.82)	3.53	**0.002**	11.51 (2.86, 20.15)	2.61	**0.013**	3.17 (-7.78, 14.13)	0.57	0.574	6.61 (0.91, 12.30)	2.27	**0.029**
**Digit Span Forward**	3.37 (-1.54, 8.28)	1.34	0.187	3.88 (-0.20, 7.95)	1.86	0.070	5.18 (-0.41, 10.77)	1.82	0.078	2.08 (-2.14, 6.29)	0.966	0.340	0.82 (-4.00, 5.63)	0.33	0.742	1.08 (-1.64, 3.80)	0.78	0.442
**TMT-A**	1.74 (-22.98, 26.45)	0.14	0.891	-4.20 (-25.45, 17.05)	-0.39	0.700	4.68 (-24.40, 33.76)	0.32	0.754	-0.65 (-21.72, 20.42)	-0.061	0.952	2.32 (-21.52, 26.15)	0.19	0.850	-4.83 (-19.58, 9.93)	-0.64	0.525

Abbreviations: CI = 95% confidence interval; EYO = estimated years to symptom onset; MC = ADAD mutation carrier; noMC = ADAD mutation non-carrier : RAVL = Rey Auditory Verbal Learning; RO = Rey-Osterrieth Complex Figure; TMT = Trail Making Test

Supplementary Table 10 Longitudinal associations between subcortical [18F]FDG PET and cognitive test scores across estimated year to symptom onset in *APP* carriers compared to *PSEN1* carriers

	Thalamus **PSEN1-APP*			Hippocampus **PSEN1-APP*			Amygdala **PSEN1-APP*			Caudate**PSEN1-APP*			Pallidum **PSEN1-APP*			Putamen **PSEN1-APP*		
**Outcome**	**Estimate (C.I.)**	***t*-value**	***P*-value**	**Estimate (C.I.)**	***t*-value**	***P*-value**	**Estimate (C.I.)**	***t*-value**	***P*-value**	**Estimate (C.I.)**	***t*-value**	***P*-value**	**Estimate (C.I.)**	***t*-value**	***P*-value**	**Estimate (C.I.)**	***t*-value**	***P*-value**
**RAVL learning**	0.66 (-2.29, 3.62)	0.44	0.662	-2.82 (-8.14, 2.49)	-1.04	0.304	0.91 (-5.13, 6.95)	0.29	0.769	0.49 (-1.37, 2.35)	0.51	0.609	-2.83 (-6.53, 0.88)	-1.49	0.142	-2.78 (-5.96, 0.41)	-1.71	0.094
**RAVL retention**	4.58 (-1.73, 10.89)	1.42	0.181	-4.20 (-15.00, 6.61)	-0.76	0.453	0.16 (-10.48, 10.79)	0.03	0.977	2.68 (-1.04, 6.41)	1.41	0.173	-1.89 (-8.58, 4.81)	-0.55	0.585	-0.26 (-8.05, 7.54)	-0.06	0.949
**RO retention**	3.88 (0.34, 7.42)	2.15	**0.038**	-2.24 (-9.02, 4.53)	-0.65	0.520	-2.80 (-9.56, 3.96)	-0.81	0.423	2.20 (0.04, 4.36)	1.99	**0.052**	-0.54 (-4.57, 3.50)	-0.26	0.796	-1.53 (-5.70, 2.64)	-0.72	0.476
**Digit Symbol**	5.14 (2.09, 8.19)	3.30	**0.002**	-3.07 (-9.27, 3.14)	-0.97	0.338	2.22 (-3.99, 8.43)	0.70	0.487	3.82 (1.76, 5.88)	3.64	**8.88e-04**	1.18 (-2.85, 5.20)	0.57	0.569	-0.90 (-4.73, 2.93)	-0.46	0.649
**TMT-B**	9.38 (1.50, 17.26)	2.33	**0.025**	9.83 (-7.51, 27.17)	1.11	0.274	20.78 (3.95, 37.61)	2.42	**0.020**	9.57 (6.48, 12.66)	6.08	**4.47e-07**	0.15 (-10.36, 10.67)	0.03	0.977	3.05 (-6.57, 12.66)	0.62	0.538
**Digit Span Forward**	0.26 (-3.92, 4.44)	0.12	0.905	4.78 (-2.79, 12.35)	1.24	0.225	3.16 (-5.41, 11.73)	0.72	0.475	1.96 (-0.81, 4.74)	1.39	0.174	4.06 (-1.06, 9.17)	1.55	0.129	3.67 (-0.85, 8.19)	1.59	0.123
**TMT-A**	10.07 (-11.06, 31.20)	0.93	0.355	-45.49 (-80.40, -10.58)	-2.55	**0.014**	-15.88 (-56.47, 24.72)	-0.77	0.448	-1.19 (-15.32, 12.94)	-0.17	0.869	1.37 (-27.26, 29.99)	0.09	0.926	-6.40 (-30.45, 17.65)	-0.52	0.604

Abbreviations: CI = 95% confidence interval; EYO = estimated years to symptom onset; MC = ADAD mutation carrier; noMC = ADAD mutation non-carrier : RAVL = Rey Auditory Verbal Learning; RO = Rey-Osterrieth Complex Figure; TMT = Trail Making Test

Supplementary Table 11 Longitudinal associations between plasma glial fibrillary acidic protein (GFAP) and [18F]FDG PET across estimated year to symptom onset in ADAD mutation carriers compared to non-carries.

	**GFAP*noMC-MC**			**GFAP*noMC-*PSEN1***			**GFAP*noMC-*APP***			**GFAP**APP-PSEN1***		
**Outcome**	**Estimate (C.I.)**	***t*-value**	***P*-value**	**Estimate (C.I.)**	***t*-value**	***P*-value**	**Estimate (C.I.)**	***t*-value**	***P*-value**	**Estimate (C.I.)**	***t*-value**	***P*-value**
**Temporoparietal**	-3.55e-02 (-0.12, 0.05)	-0.84	0.404	-1.17e-02 (-0.10, 0.08)	-0.26	0.795	-1.31e-01 (-0.22, -0.04)	-2.74	**0.008**	7.65e-02 (-0.004, 0.16)	1.87	0.076
**Frontal**	-6.92e-02 (-0.17, 0.03)	-1.41	0.164	-4.661e-02 (-0.15, 0.06)	-0.89	0.375	-1.40e-01 (-0.25, -0.03)	-2.51	**0.015**	0.06 (-0.02, 0.13)	1.42	0.167
**Temporal**	-2.60e-02 (-0.09, 0.04)	-0.77	0.444	-1.00e-02 (-0.08, 0.06)	-0.27	0.787	-9.246e-02 (-0.17, -0.02)	-2.34	**0.023**	3.55e-02 (-0.02, 0.09)	1.19	0.246
**Parietal**	-4.22e-02 (-0.15, 0.06)	-0.78	0.438	-2.66e-03 (-0.12, 0.11)	-0.05	0.964	-1.43e-01 (-0.26, -0.02)	-2.31	**0.025**	9.36e-02 (-0.03, 0.22)	1.45	0.163
**Occipital**	-4.19-03 (-0.11, 0.10)	-0.08	0.939	-9.95e-03 (-0.13, 0.11)	-0.17	0.869	-1.00e-01 (-0.23, 0.03)	-1.57	0.123	7.24e-02 (-0.05, 0.19)	1.17	0.251
**Posterior cingulate**	-1.12e-01 (-0.26, 0.04)	-1.49	0.142	-1.59e-02 (-0.16, 0.13)	-0.21	0.834	-0.68e-01 (-0.43, -0.11)	-3.31	**0.002**	1.91e-01 (-0.03, 0.41)	1.70	0.101
**Thalamus**	-9.323e-03 (-0.14, 0.12)	-0.14	0.886	8.02e-02 (-0.05, 0.21)	1.21	0.232	-1.33e-01 (-0.27, 0.01)	-1.87	0.067	2.54e-01 (0.14, 0.37)	4.45	**0.0002**
**Hippocampus**	-1.24e-02 (-0.07, 0.05)	-0.41	0.687	4.30e-02 (-0.02, 0.10)	1.36	0.181	-1.06e-01 (-0.17, -0.04)	-3.04	**0.004**	9.70e-02 (0.01, 0.18)	2.19	**0.038**
**Amygdala**	-6.36e-02 (-0.13, 0.01)	-1.82	0.076	9.20e-03 (-0.06, 0.08)	0.27	0.789	-1.50e-01 (-0.22, -0.08)	-4.08	**0.0002**	1.22e-01 (0.06, 0.18)	3.94	**0.0005**
**Caudate**	-6.37e-02 (-0.18, 0.06)	-1.05	0.300	1.17e-02 (-0.11, 0.14)	0.18	0.854	-1.55e-01 (-0.29, -0.02)	-2.28	**0.027**	1.27e-01 (0.03, 0.23)	2.48	**0.020**
**Pallidum**	-3.96e-02 (-0.13, 0.05)	-0.86	0.394	-3.69e-02 (-0.14, 0.06)	-0.74	0.466	-8.99e-02 (-0.20, 0.02)	-1.57	0.123	1.16e-01 (0.01, 0.22)	2.08	**0.048**
**Putamen**	-5.76e-03 (-0.13, 0.12)	-0.09	0.928	3.88e-03 (-0.13, 0.14)	0.06	0.955	-1.21e-01 (-0.26, 0.02)	-1.65	0.104	1.27e-01 (0.02, 0.23)	2.31	**0.028**

Abbreviations: CI = 95% confidence interval; EYO = estimated years to symptom onset; MC = ADAD mutation carrier; noMC = ADAD mutation non-carrier : RAVL = Rey Auditory Verbal Learning; RO = Rey-Osterrieth Complex Figure; TMT = Trail Making Test

Supplementary Table 12 Longitudinal associations between plasma glial fibrillary acidic protein (GFAP) and cognition in ADAD mutation carriers compared to non-carries.

	GFAP*noMC-MC			GFAP*noMC-*PSEN1*			GFAP*noMC-*APP*			GFAP**APP-PSEN1*		
**Outcome**	**Estimate (C.I.)**	***t*-value**	***P*- value**	**Estimate (C.I.)**	***t*-value**	***P*-value**	**Estimate (C.I.)**	***t*-value**	***P*-value**	**Estimate (C.I.)**	***t*-value**	***P*-value**
**RAVL learning**	-0.94 (-1.79, -0.09)	-2.16	**0.035**	-0.40 (-1.26, 0.46)	-0.91	0.367	-1.13 (-2.04, -0.21)	-2.401	**0.020**	0.14 (-0.82, 1.09)	0.23	0.784
**RAVL retention**	-0.74 (-1.81, 0.32)	-1.36	0.180	-0.47 (-1.65, 0.71)	-0.78	0.439	-0.92 (-2.18, 0.33)	-1.442	0.156	0.38 (-1.07, 1.39)	0.26	0.799
**RO retention**	-0.70 (-1.61, 0.21)	-1.51	0.140	-0.08 (-1.05, 0.90)	-0.15	0.878	-1.62 (-2.74, -0.51)	-2.851	**0.006**	0.06 (-1.06, 1.19)	0.11	0.912
**Digit Symbol**	-0.40 (-0.88, 0.07)	-1.65	0.106	-0.03 (-0.52, 0.47)	-0.11	0.916	-0.93 (-1.51, -0.35)	-3.126	**0.003**	0.36 (-0.46, 1.17)	0.86	0.397
**TMT-B**	-0.54 (-1.30, 0.23)	-1.38	0.174	-0.13 (-0.89, 0.63)	-0.34	0.737	-1.29 (-2.22, -0.35)	-2.694	**0.010**	0.32 (-0.69, 1.34)	0.62	0.539
**Digit Span Forward**	0.22 (-0.47, 0.92)	0.63	0.532	0.45 (-0.33, 1.22)	1.13	0.267	0.04 (-0.84, 0.92)	0.085	0.933	0.73 (-0.52, 1.98)	1.14	0.266
**TMT-A**	-0.19 (-1.17, 0.78)	-0.39	0.701	0.51 (-0.44, 1.45)	1.05	0.304	-1.62 (-2.85, -0.39)	-2.583	**0.015**	1.61 (0.22, 2.99)	2.28	**0.036**

Abbreviations: CI = 95% confidence interval; EYO = estimated years to symptom onset; MC = ADAD mutation carrier; noMC = ADAD mutation non-carrier : RAVL = Rey Auditory Verbal Learning; RO = Rey-Osterrieth Complex Figure; TMT = Trail Making Test
